# The geography of imported malaria to non-endemic countries: a meta-analysis of nationally reported statistics

**DOI:** 10.1016/S1473-3099(16)30326-7

**Published:** 2017-01

**Authors:** Andrew J Tatem, Peng Jia, Dariya Ordanovich, Michael Falkner, Zhuojie Huang, Rosalind Howes, Simon I Hay, Peter W Gething, David L Smith

**Affiliations:** a WorldPop, Department of Geography and Environment, University of Southampton, Southampton, UK; b Flowminder Foundation, Stockholm, Sweden; c Faculty of Geo-information Science and Earth Observation (ITC), University of Twente, Enschede, Netherlands; d Department of Geography, University of Florida, Gainesville, FL, USA; e Division of Infectious Diseases, Key Laboratory of Surveillance and Early-warning on Infectious Disease, Chinese Center for Disease Control and Prevention, Beijing, China; f Spatial Ecology and Epidemiology Group, Department of Zoology, University of Oxford, Oxford, UK; g Centre for Global Health and Diseases, Case Western Reserve University, Cleveland, OH, USA; h Institute for Health Metrics and Evaluation, University of Washington, Seattle WA, USA; i Oxford Big Data Institute, Li Ka Shing Centre for Health Information and Discovery, University of Oxford, Oxford, UK

## Abstract

**Background:**

Malaria remains a problem for many countries classified as malaria free through cases imported from endemic regions. Imported cases to non-endemic countries often result in delays in diagnosis, are expensive to treat, and can sometimes cause secondary local transmission. The movement of malaria in endemic countries has also contributed to the spread of drug resistance and threatens long-term eradication goals. Here we focused on quantifying the international movements of malaria to improve our understanding of these phenomena and facilitate the design of mitigation strategies.

**Methods:**

In this meta-analysis, we studied the database of publicly available nationally reported statistics on imported malaria in the past 10 years, covering more than 50 000 individual cases. We obtained data from 40 non-endemic countries and recorded the geographical variations.

**Findings:**

Infection movements were strongly skewed towards a small number of high-traffic routes between 2005 and 2015, with the west Africa region accounting for 56% (13 947/24 941) of all imported cases to non-endemic countries with a reported travel destination, and France and the UK receiving the highest number of cases, with more than 4000 reported cases per year on average. Countries strongly linked by movements of imported cases are grouped by historical, language, and travel ties. There is strong spatial clustering of plasmodium species types.

**Interpretation:**

The architecture of the air network, historical ties, demographics of travellers, and malaria endemicity contribute to highly heterogeneous patterns of numbers, routes, and species compositions of parasites transported. With global malaria eradication on the international agenda, malaria control altering local transmission, and the threat of drug resistance, understanding these patterns and their drivers is increasing in importance.

**Funding:**

Bill & Melinda Gates Foundation, National Institutes of Health, UK Medical Research Council, UK Department for International Development, Wellcome Trust.

## Introduction

Tackling malaria remains a high priority internationally, with elimination and eradication back on the global agenda.^1^ In the past century, more than 50 countries have succeeded in eliminating the disease. Nevertheless, although malaria is no longer endemic in these nations, increasing travel to endemic areas in recent decades means that the malaria-endemic world is becoming increasingly connected by population movements,^2^ and that imported malaria cases remain common.^3^ Such cases continue to pose challenges for diagnosis and management, with malaria remaining an infrequently encountered disease for many physicians in non-endemic areas,^4^ where it can be expensive to treat^5^ and result in high mortality.^6^ Moreover, with anopheles vectors still present in many non-endemic countries, imported cases can also cause secondary transmission,^7^ although the chances of resumption of endemic transmission are very small.^8^ Finally, although the effects of imported malaria on malaria-free countries are problematic, data on the features of imported cases can also provide valuable information about both the epidemiology of malaria in endemic regions where surveillance systems are weak, and on how malaria moves around the world.^2^

The timing, number, and origin of imported malaria cases into non-endemic regions vary by country and are a function of several factors including the transmission intensity of the origin location, the number of people visiting that location, the activities undertaken in the location, and prophylaxis availability and adherence.^3^, ^9^, ^10^, ^11^ Depending on the country, some demographic groups have substantially higher infection rates. For instance, malaria imported to Europe is often reported in travellers returning from (or migrants coming from) endemic areas and migrants living in Europe returning from visiting friends and relatives, with children who are visiting friends and relatives being particularly at risk.^12^

Information about imported malaria is to an extent captured by national authorities where, for most high-income countries, malaria is a notifiable disease. However, under-reporting is probably common; for instance, WHO reported that 6244 cases of malaria were imported to Europe in 2010, but the true number might be six times higher.^13^ Such deficiencies have prompted the initiation of surveillance networks such as GeoSentinel^14^ and EuroTravNet,^15^, ^16^ which now play a key part in the surveillance of imported malaria, in the identification of changing trends in malaria importation, in tracking drug-resistance patterns, and in establishing the changing profile of malaria risk at traveller destinations. Nevertheless, nationally reported data continue to be widely collected and still provide valuable information about the trends, composition, and drivers of imported malaria for most non-endemic countries, with annual data compilations and analyses of statistics providing the main source of information guiding national policies on imported malaria. However, a contemporary global assembly of such nationally reported data, and assessment of patterns and variations has not previously been undertaken.

**Research in context**

**Evidence before this study**

The rise of air travel in the past century has resulted in a highly interconnected world, where geographical distance is becoming less of a barrier to pathogen dispersal. Malaria-free countries that were once endemic to the disease still report thousands of cases every year through importation, resulting in deaths, health system burden, and occasional secondary transmission. The disease is notifiable in most high-income countries, providing data on case numbers and characteristics to direct mitigation strategies. Multiple individual studies at national scales over the past decade have highlighted the substantial heterogeneities in imported malaria numbers, rates, and risks that exist across geographies, demographics, and malaria species, but have not been brought together in a single study.

**Added value of this study**

This study reports the first global collection of nationally reported imported malaria statistics in 20 years. Moreover, it presents the first global assessment of the geographical features of imported malaria, including patterns of flows, connectivity, species distributions, and diagnostic capacities. Our findings show how limits to malaria dispersal remain and how clear patterns in movements exist that have never been quantified before, with the architecture of the air network, historical ties, demographics of travellers, and malaria endemicities all contributing to highly heterogeneous patterns of numbers, routes, and species compositions of parasites transported.

**Implications of all the available evidence**

With global malaria eradication on the international agenda, the threat of spreading drug resistance, and the continued burden of imported cases to non-endemic countries, understanding and measuring the patterns of malaria connectivity and their drivers is increasing in importance for designing mitigation, control, and elimination strategies. Prioritising surveillance and control efforts to high-traffic routes and highly connected locations, as well as coordinating elimination efforts around highly connected regional groupings of countries is likely to be the most effective and efficient approach.

Here, we describe the first global assembly of nationally reported imported malaria data in 20 years,^11^ and the geographic analysis of these data from 40 non-endemic countries in the past 10 years. We map the system of transmission from endemic to non-endemic areas to provide insights into the underlying dynamics of the system. Exploration of the driving factors behind these patterns is beyond the scope of this study. We examine the rates of flow of cases from endemic to non-endemic countries and their inherent spatial patterns. Moreover, we map the species composition of cases by sending and receiving regions. Finally, we discuss candidate factors that shape the recorded patterns and likely future trends.

## Methods

Full details of the process of constructing a library of imported malaria statistics and data extractions are provided in appendix (pp 2–8). Here we provide brief details of the steps taken in assembly and analysis of the data.

### National imported malaria statistics

Most non-endemic countries compile their notified cases of malaria into annual summary reports. These reports were assembled from the national laboratories and agencies that compile imported malaria statistics for as many years as available (appendix). We complemented these data with searches on PubMed, Web of Science, Google Scholar, and standard Google search for “imported malaria” and the name of the non-endemic country in question, in both English and the primary language of the country where this was not English. These searches identified a set of additional academic papers and reports documenting imported cases. For each country, where available, we extracted data on the numbers of confirmed cases reported, the year, their likely origin regions or countries, the species of parasite, and the method of diagnosis.

### Data processing

We constructed a set of broad rules to facilitate data summarisation, exclusion, and processing. These were based on achieving a balance between maintaining a wide representation of data from several countries, time periods, and sources, and implementing some quality control to ensure comparability between datasets and avoid double-counting. We minimised double-counting through examination of data obtained from academic publications—if they were obtained from nationally reported statistics covering the same period as already extracted from national laboratory reports, then the data were not included in analyses. Throughout, we prioritised statistics from national agencies over academic papers, which were used as supplemental data sources to cover missing periods. We did different hierarchies of analysis to enable presentations of outputs where data inclusion criteria were relaxed to enable comparisons across many countries, and criteria were tightened or data were aggregated to facilitate the production of more robust, but less detailed, conclusions.

Although the datasets assembled extended from 1960 to the end of 2015, to obtain a contemporary picture while still including a large number of countries and regions, analyses were undertaken only for the most recent 10 years of data. Therefore, data were restricted to 2005–15, using an annual mean of cases across the full 10 years when available, although for some countries, data were only available for less than 5 years of this period. For each endemic exporting country, we aggregated all reported annual mean case numbers exported to non-endemic reporting countries to obtain estimates of the proportions of each parasite species (or mixed infections, when documented as such) exported. Although this averaging masked temporal trends in the data, clear trends over time were not apparent for most countries, and in view of the gaps in publicly available data (appendix), this time window facilitated the inclusion of many more countries than a more constrained one. We constructed origin–destination matrices for the average number of cases per year imported from endemic to non-endemic countries. Many data sources reported exported cases only by large regions, therefore we also constructed a regional version of this matrix to enable the inclusion of more data and thus identify geographical patterns more robustly. We also analysed these data to estimate the aggregate malaria species compositions being exported from endemic countries and imported to non-endemic countries. Where species breakdowns of imported cases were reported, they were aggregated and summarised across the reporting period.^17^ Similar to the origin–destination matrices, data for species composition for many countries were reported only by origin region; thus data were also aggregated by region to provide larger sample sizes and thus more confidence in estimates of differences between regions by composition.

### Network community detection

Communities in a network reflect a group of nodes that are densely connected and separated from the other nodes in the network, and thus they share common properties and have similar roles within the network. By mapping communities on the imported malaria network defined here, we aimed to identify groups of countries that show strong links in terms of movements of infected travellers. Newman and Girvan^17^ define a modularity score, which is a measure of the strength of a division of a network into communities (groups of countries in this case). The analysis uses a multilevel algorithm for community detection,^18^ which uses an iterative approach that merges communities to maximise the modularity.

### Additional datasets

The construction of modelled global *Plasmodium falciparum*^19^ and *Plasmodium vivax*^20^ parasite prevalence maps enabled simple comparisons to be made with the imported malaria statistics. The datasets were obtained from the Malaria Atlas Project and summarised to a national level using gridded population data from the WorldPop project and the Global Rural Urban Mapping Project to produce a population-weighted mean *P falciparum* and *P vivax* prevalence for each country. Additionally, we obtained bilateral data^21^ for migrations between each pair of endemic and non-endemic countries to enable further comparisons to be made with the number of cases of imported malaria.

### Role of the funding source

The funders of the study had no role in study design, data collection, data analysis, data interpretation, or writing of the report. The corresponding author had full access to all the data in the study and had final responsibility for the decision to submit for publication.

## Results

**Figure 1:**
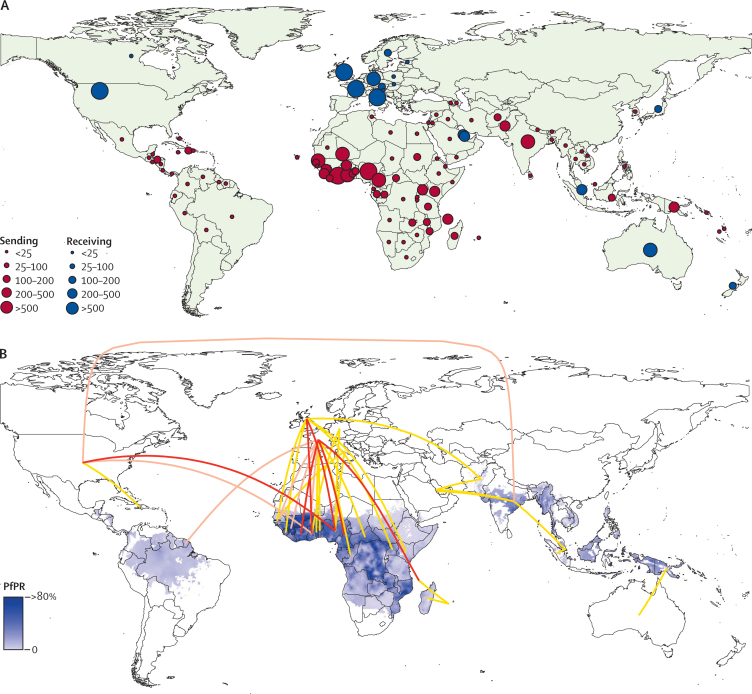
Origins, destinations, and flows of imported cases of malaria from endemic to non-endemic countries (A) Of the non-endemic countries that reported the origin country of imported cases, the average annual number of malaria cases (all species) between 2005 and 2015 exported from endemic to non-endemic countries (red) and imported cases to non-endemic from endemic countries (blue). (B) Malaria endemic to non-endemic country connectivity through cases imported to the non-endemic country. Of the non-endemic countries that reported the origin country of imported cases, the average annual number of malaria cases (all species) between 2005 and 2015 moving from endemic to non-endemic country pairs are mapped as flow lines. Only average annual flows of >50 cases are mapped, with >200 in red, 100–200 in pink, and 50–100 in yellow. The flow lines are overlaid on a map of *Plasmodium falciparum* prevalence.^19^

**Figure 2:**
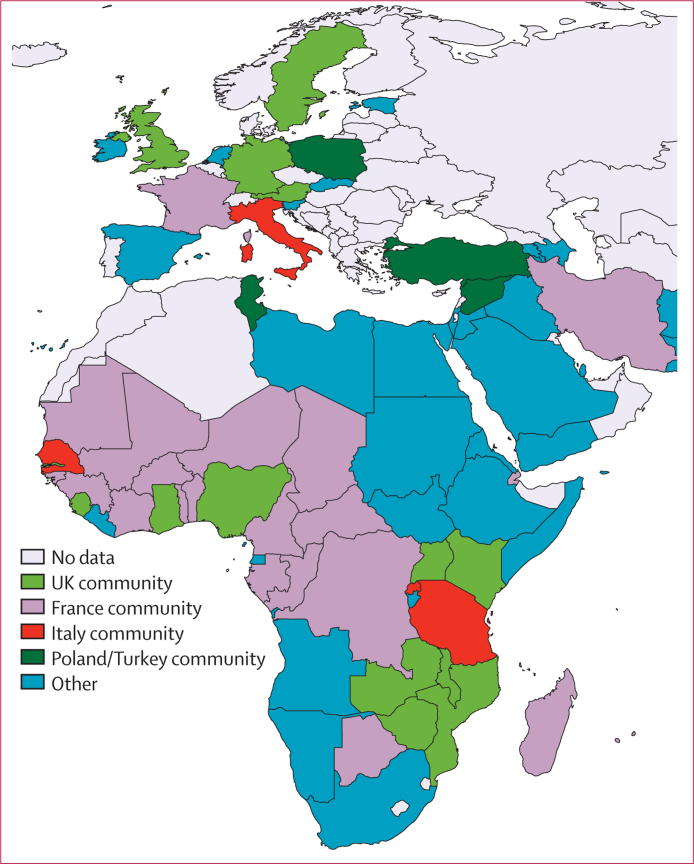
Results of community detections on the network of malaria endemic–non-endemic imported malaria pairs Countries mapped in the same colour belong to a unique community, with imported malaria case movements being larger within the communities than between them. Fewer cases in Asia and the Americas had origin–destination information available, making the community detection results less robust. Moreover, with substantially fewer non-endemic countries outside of Europe with strong reporting, analyses simply show the regions as homogeneous single communities.

The movement of people with malaria in 2005–15 followed specific routes (figure 1). Of those cases with origin location recorded between 2005 and 2015, more cases were reported in France (2169 cases per year on average) and the UK (n=1898) than any other country (figure 1A), with the USA (n=1511), Italy (n=637), and Germany (n=401) close behind. Most (22 946/24 941 [92%]) exported cases to non-endemic countries originate in west Africa (13 947 [56%]), India (4988 [20%]), east Africa (3242 [13%]), and Papua New Guinea (748 [3%]; figure 1A). Figure 1B shows that the connections between the UK and west Africa, and between France and west Africa are the strongest in terms of annual average numbers of cases moving from endemic to non-endemic countries (2492 cases on average per year, 2005–15), but that many other routes produce an annual average of more than 50 cases reported in non-endemic countries. These include the movements between the USA and India (149 cases per year on average), the USA and west Africa (n=716), the USA and Haiti (n=52), Australia and Papua New Guinea (n=97), and the UK and Pakistan (n=69). By defining the origin–destination pairs of endemic–non-endemic countries and the cases originating and reported in each as a weighted network, the community detection analyses identified sections of this global imported malaria flow matrix that were more strongly connected than others. Although the matrix is incomplete, with country-level imported malaria data unavailable for some non-endemic countries, it is clear that the structure of these mapped network communities is not mainly geographically determined (figure 2), with historical, economic, language, and cultural ties evident. For example, the UK community includes the English-speaking African nations connected to the UK that were its former colonies (including Nigeria, Ghana, Kenya, Uganda, and The Gambia), and French colonial ties are also evident (including Mali, Niger, Chad, CÔte d'Ivoire, Burkina Faso, Benin, Togo, and Madagascar).

**Figure 3:**
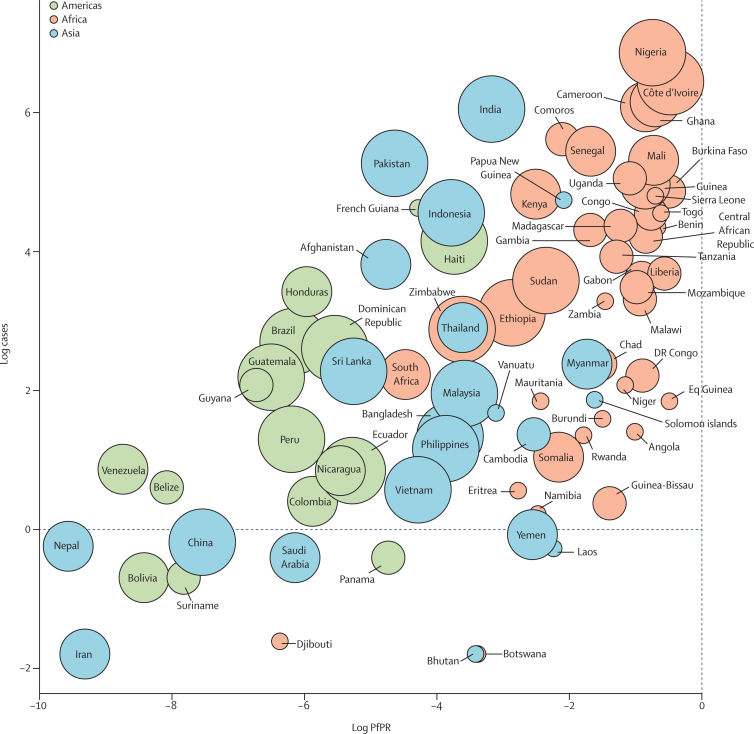
*Plasmodium falciparum* prevalence in 2010 versus average annual number of *P falciparum* cases imported to non-endemic countries for all endemic countries, 2005–15 The circles are coloured by region (green=Americas, pink=Africa, blue=Asia), and their sizes correspond to numbers of outgoing migrants to non-endemic countries. Linear model fit to *P faciparum* parasite rate (PfPR) against *P faciparum* cases: r2=0·32; p<0·01.

Clear associations exist between the average annual number of outgoing *P falciparum* cases from endemic to non-endemic countries, *P falciparum* prevalence in the endemic countries, and the migration flows to non-endemic countries (figure 3). Moreover, the countries with the most migrants residing in non-endemic countries (eg, India, Pakistan, and Nigeria) typically fall further towards the upper-left side of the plot, as larger numbers of travellers (particularly those visiting friends and relatives in endemic countries) are related to larger numbers of imported cases to non-endemic countries. Although there are associations between the numbers of cases and both malaria prevalence in endemic countries and numbers of migrants in non-endemic countries, many other factors play a part, including demographics, levels of prophylaxis and protection use, and travel activities.

**Figure 4:**
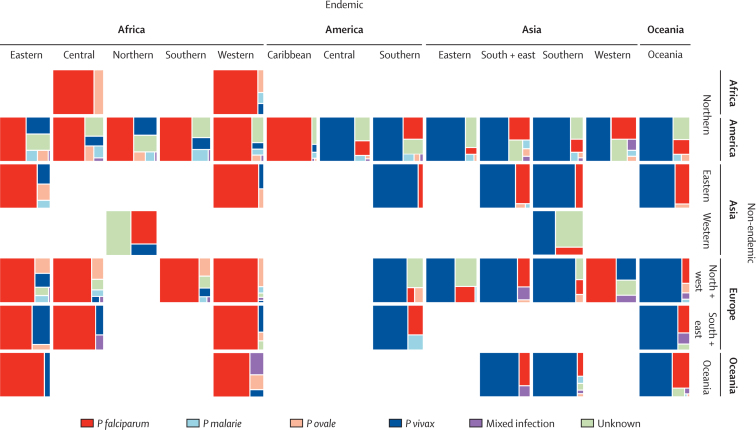
Pooled data for imported malaria species breakdown to non-endemic regions from endemic ones The figure shows proportions of total imported case numbers, rather than absolute numbers, which are shown in figure 1A.

**Figure 5:**
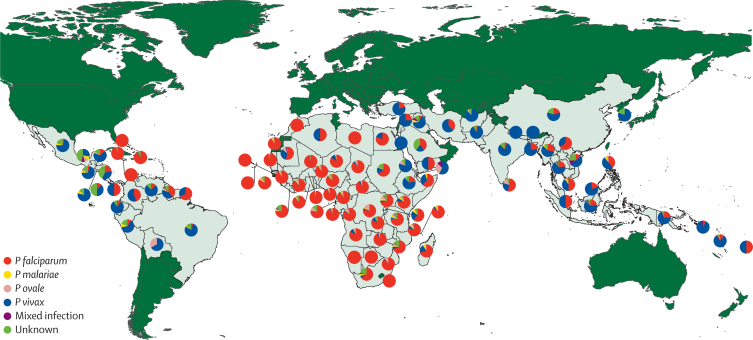
Species composition of reported imported malaria cases to non-endemic countries mapped by endemic country of origin of the cases

We analysed the species compositions of cases reported in non-endemic countries by region (figure 4) and nation (figure 5 and table). Reports of cases in non-endemic countries often present species type broken down by origin region rather than country, thus initially we did the species composition analyses at pooled regional level to ensure larger and more stable sample sizes (figure 4). The results emphasise the large variation in the species composition of malaria cases travelling between the different regions of the world. Although these outputs represent a pooling of data of varying numbers, time periods, treatment-seeking behaviours, and diagnostic capacities, the clear and geographically consistent patterns suggest a robustness in the outputs. The dominance of *P falciparum* from African and Caribbean sources (mean percentage of cases across regions 74·4%) compared with those originating in Central and South America (13·1%) and Asia and Oceania (17·6%) is clear, although no single species in any region has total dominance. This finding is also reflected at national levels (figure 5), with strong geographically coherent patterns recorded, but also a mixed picture in many places, especially in southeast Asia and central America. The species compositions of cases received in each non-endemic country (figure 4 and table) are indicative of each country's connections to endemic regions. For example, the high *P falciparum* percentage for France results from its strong ties to west Africa (figure 1). European proximity and ties to Africa result in more cases of *P falciparum* (mean percentage of cases of 65·8%) than in the Americas (41·7%) or the Asia-Pacific region (32·9%), although a divide is clear, with higher proportions of *P vivax* in eastern compared with western Europe evident (figure 4). Finally, analyses of diagnostic capacities in European countries (appendix pp 9, 10) highlight the growth in capacities in the past decade, with an increasing use of PCR and rapid tests. However, substantial geographical differences remain, with the range of methods and their reporting higher in western Europe than eastern Europe.

## Discussion

The substantial growth in the reach and rates of human travel, in particular the air traffic network, in recent decades, has had a major effect on global disease epidemiology, including malaria.^9^ Rising rates of travel to and from endemic areas has resulted in imported malaria being frequently reported in malaria-free countries, with occasional secondary transmission.^7^ However, this travel expansion has not been ubiquitous, with historical and economic ties driving growth along certain routes far more than others, and resulting in uneven malaria movement.^2^ Moreover, substantial investment in malaria control in recent decades has resulted in overall decreased prevalence in endemic areas, with some areas noting especially large decreases,^22^ further contributing to variations in importation to non-endemic countries. Here, we have presented unique analyses of a global assembly of publicly available contemporary data for the national reporting of imported malaria to capture these variations and quantify the broad geographic features.

We noted clear and consistent patterns despite differences in data quality, completeness, and temporality, and data being indicative of the different surveillance systems and diagnostic capacities of the reporting countries (appendix pp 9, 10). Our results underline the substantial geographical heterogeneities that exist in reported malaria case numbers and compositions in non-endemic countries. Moreover, certain routes from endemic to non-endemic countries carry substantially more infections than others, with evidence of tight couplings that reflect historical ties. These communities of countries can serve to guide surveillance, develop mitigation strategies, and highlight likely routes of drug-resistant malaria movement.^23^ The tight coupling of locations also highlights risks for secondary transmission following imported cases, such as through immigrant labour in the Middle East^24^ or Chinese labourers returning from Africa.^25^ Further, the species compositions highlight *P vivax, P ovale*, and *Plasmodium malariae* as potential malaria parasites in areas of the world where they are rarely considered, such as much of Africa. Coupled with policy shifts towards species-specific diagnostics and reporting, this finding could prompt a robust assessment of the more neglected non-falciparum parasites that can still cause severe clinical illness and require specific control interventions.

We have endeavoured to minimise the uncertainties and errors that arise through the analysis of data from such a large range of sources. However, many factors, including the opportunistic and highly varied nature of the available data, affect our ability to compare between countries and draw precise conclusions. First, the data represent a small proportion of a possibly larger pool of cases, with some estimates suggesting that national statistics might capture just one-sixth of all imported cases.^13^ Variations in health system reporting mechanisms and diagnostic capacities between countries also probably mean that some countries capture more cases than others, some cities and regions within countries capture more than others, and some countries have a greater capacity to undertake reliable speciation through using PCR or having more experienced and well trained microscopists. Microscopic examination is widely available in most non-endemic countries (appendix pp 9, 10); however, misdiagnoses or late diagnoses can still be common because of the failure of medical personnel to relate the febrile symptoms to a disease that is rarely reported in their region. Malaria symptoms are non-specific and cannot easily be distinguished from other febrile disorders on clinical grounds alone. Moreover, changes in reporting standards, practices, and capacity over time within nations can affect the comparability of data over time, and affect outcomes and representativeness when data are pooled over many years. Microscopic diagnosis is often slow and inaccurate in non-specialised laboratories.^26^, ^27^ In some cases, molecular assays can become insufficient to make a correct diagnosis, especially to detect all species in mixed infections or in cases when parasitaemia is low, which is often the case in non-immune patients who complied with chemoprophylaxis.

Moreover, one or more species in mixed species infections are easily overlooked, and some species are more difficult to classify than others, with, for example, morphological similarities between *P vivax* and *P ovale* potentially a source of misclassification.^28^ In relation to these classification challenges, the confidence in the reports regarding imported malaria varies between studies depending on the method used to detect the infection. The large percentages of unknown malaria types for some countries are likely to be indicative of a lack of diagnostic capacity. National health statistics often do not report the techniques used, and therefore it is necessary to refer to the academic publications describing the summarised national data in which this information is provided to assess the overall precision of the diagnosis at the country level. Finally, intervention scale-up,^22^ urbanisation,^29^ changing wealth,^30^ and improved health systems are all likely to have affected the prevalence and species composition of malaria in endemic regions in the past decade, subsequently affecting the comparability of imported case data in non-endemic regions between years. Nevertheless, the clear, consistent, and coherent patterns we recorded within regions and between countries and the congruence with results through dedicated surveillance networks,^3^, ^14^, ^15^, ^16^ suggest that the data presented here form a representative sample.

Second, several factors relating to differences in traveller type and activity between countries contribute to the representativeness, comparability between nations, and uncertainties in outputs. Rates of chemoprophylaxis, prescription, use, and antimalarial adherence vary by country and by demographic group,^31^, ^32^ as does the use of protective measures while travelling.^33^ Further, the demographics and ethnic composition of traveller groups vary by country; for example, nations that have large migrant populations originating from endemic countries probably contribute to more cases arising from those visiting friends and relatives.^6^ The proportion of imported malaria cases due to migrants in Europe has increased in the past 15 years,^34^ with those visiting friends and relatives travelling to endemic areas of Africa more than eight times more likely to be diagnosed with malaria compared with tourists,^35^ and their children being especially at risk.^36^ Activities in endemic regions might also contribute to differences recorded; for instance, people travelling to urban areas and staying in hotels are likely to be at lower risk. Differences in demographics and health systems also translate to differences in treatment seeking as well as whether case importation occurs principally through visitors or travelling residents. Some demographic groups are more likely to seek treatment than others for travel-related health issues.^34^

Our study is an ongoing effort. Summaries of national malaria surveillance data are not made publicly available for all countries and years and many additional relevant datasets probably remain unpublished, so we welcome input from those who have access to datasets not included here to enable continual updates. Our study provides a global picture of malaria importation to non-endemic countries, but does not extend to exploration of the driving factors behind these patterns. However, our future work will focus on building datasets and a modelling framework for understanding what drives the patterns noted here.

The associations with malaria endemicity and migration flows suggest two key drivers, but further data for travel patterns and volumes, malaria transmission, demographics, health system efficiency, diagnostic capacities, treatment-seeking behaviours, and prophylaxis compliance and availability, among other factors, need to be collated to better explain and model the malaria importation patterns recorded, with a goal of predictive modelling. Further, such analyses could be extended to other commonly imported infectious diseases^3^, ^9^, ^37^ and the effects of seasonal variations in these drivers could be incorporated.^38^, ^39^ We have focused on broad comparisons through pooling across years to provide sufficient data. This approach has probably ignored changes that have occurred across time, and future work will have to focus on augmenting and breaking these data down to explore temporal trends. Finally, our results match closely those found through analysis of data collected by surveillance networks such as GeoSentinel^14^ and EuroTravNet,^15^, ^16^ but future work should focus on undertaking quantitative comparisons.

As many countries move towards national malaria elimination, global eradication moves up the international agenda,^1^ and the threat of spreading drug resistance grows,^23^ there is an increasing focus on malaria importation and the vulnerability of countries to resurgence.^40^ This study forms part of wider efforts to understand patterns of human and malaria parasite movement and how such information can guide control and elimination efforts. Malaria parasites do not respect national borders, and with human mobility continuing to increase in its volumes and reach, increasing global connectivity,^2^ control, and treatment strategies should account for the continued globalisation of malaria.

## Figures and Tables

**Table:** Species composition of reported imported malaria cases to non-endemic countries from available data with species composition recorded between 2005 and 2015

	**Average number of cases per year**	***P falciparum***	***P vivax***	***P malariae***	***P ovale***	**Other or unknown**
Australia	222	44·8	44·4	0·0	0·0	10·8
Austria	84	56·0	34·7	2·2	0·0	7·1
Bahrain	158	14·3	85·7	0·0	0·0	0·0
Belgium	227	63·0	23·0	5·0	9·0	0·0
Bulgaria	40	68·6	23·9	1·8	2·3	3·3
Canada	20	37·9	47·1	0·7	5·7	8·6
Croatia	11	64·8	19·9	1·9	0·5	12·8
Czech Republic	20	56·7	43·3	0·0	0·0	0·0
Denmark	104	76·8	17·0	2·3	2·9	1·1
Estonia	3	61·2	25·8	1·2	1·8	10·0
Finland	31	71·7	19·2	2·3	5·7	1·1
France	2169	85·7	6·5	2·1	5·7	0·0
Germany	401	82·0	8·0	2·9	2·6	4·6
Greece	41	42·6	50·0	2·5	0·8	4·0
Hong Kong	40	21·2	52·9	8·7	8·7	8·7
Ireland	54	74·7	7·1	1·9	4·5	11·7
Israel	60	45·0	52·1	1·3	1·6	0·0
Italy	637	83·4	8·4	1·6	6·5	0·0
Japan	45	47·2	48·7	0·9	3·2	0·0
Lithuania	4	62·5	12·5	12·5	0·0	12·5
Morocco	58	86·5	1·7	1·7	10·2	0·0
Netherlands	366	75·0	25·0	0·0	0·0	0·0
New Zealand	44	33·6	58·8	1·6	2·0	4·0
Norway	49	68·1	17·0	4·0	1·4	9·4
Poland	25	71·5	21·9	1·3	2·0	3·3
Portugal	178	71·5	0·0	0·0	0·0	28·5
Qatar	146	13·7	40·0	0·0	0·0	46·3
Réunion Island	156	85·8	10·7	1·9	1·6	0·0
Romania	29	65·0	0·0	0·0	0·0	35·0
Serbia	15	62·4	16·8	0·0	0·0	20·8
Singapore	148	29·6	67·3	1·2	0·0	1·9
Slovakia	5	58·0	42·0	0·0	0·0	0·0
Slovenia	7	48·5	43·8	0·0	4·7	3·1
Spain	374	54·5	17·8	1·1	11·1	15·6
Sweden	72	63·8	27·6	1·4	7·2	0·0
Switzerland	225	80·1	11·7	3·2	3·3	1·7
UK	1898	76·4	15·1	1·8	5·9	0·7
USA	1511	46·9	16·8	2·4	2·4	31·5
