# Supplementary material for: The geography of imported malaria to non-endemic countries: a meta-analysis of nationally reported statistics
Source: Lancet Infect Dis. 2017 Jan;17(1):98–107. doi: 10.1016/S1473-3099(16)30326-7 (PMC5392593; doi:10.1016/S1473-3099(16)30326-7)
Supplement: Supplementary appendix [file mmc1.pdf]

THE LANCET Infectious Diseases

Supplementary webappendix

This webappendix formed part of the original submission and has been peer reviewed. We post it as supplied by the authors.

Supplement to: Tatem AJ, Jia P, Ordanovich D, et al. The geography of imported malaria to non-endemic countries: a meta-analysis of nationally reported statistics. *Lancet Infect Dis* 2016; published online Oct 21. http://dx.doi.org/10.1016/S1473-3099(16)30326-7.

<u>Supplemental information for '*The geography of imported malaria to non-endemic countries: a meta-analysis of nationally-reported statistics*'</u>

Additional details on the assembly and analysis of a database of publicly-available imported malaria data to non-endemic countries

An assembly of publicly available national imported malaria data from 48 non-endemic countries over the past 55 years (1960-2015) was undertaken from a combination of national agency reports and academic papers. Here, an overview of the process, as well as data sources and additional methodological details are described. Tables summarising the extracted data are provided in Supplemental file 1, which contains multiple tabs documenting the data in alphabetical order by non-endemic country, and a readme/metadata tab that provides data descriptor information. Figure S1 shows a flowchart outlining the steps taken to obtain the imported malaria data, process it and produce the analyses presented in this paper.

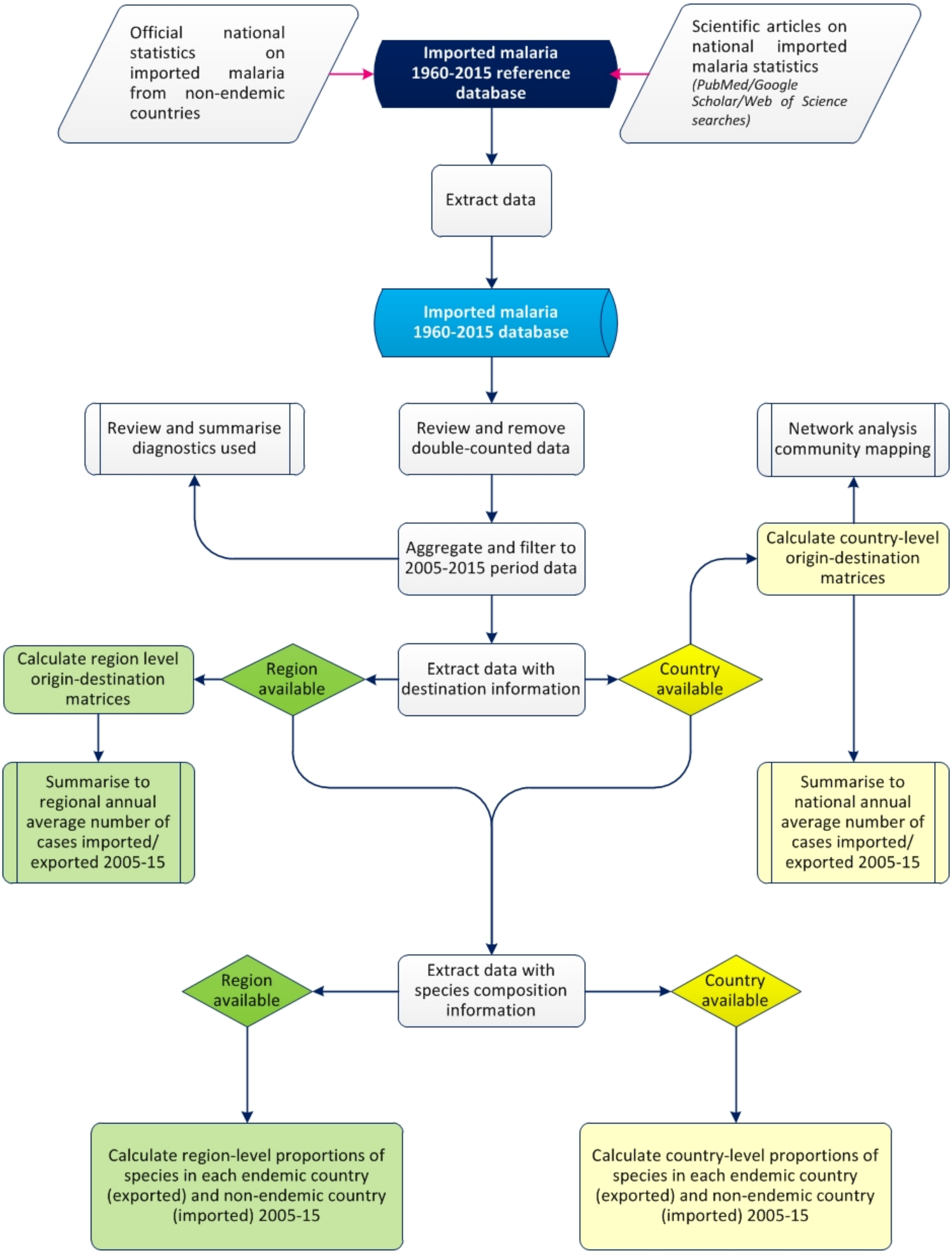

Figure S1. Flowchart documenting data gathering, processing and analysis steps undertaken

National agency reports

The majority of non-endemic countries compile their notified cases of malaria into annual summary reports. All publicly available reports were assembled from the national laboratories and agencies of non-endemic countries that compile imported malaria statistics for as many years as available. This was undertaken through initially searching the websites of reporting agencies in each non-endemic country to obtain publicly available summary statistics on imported malaria cases, including, where possible, numbers by month and year, origin countries and/or regions of cases and the species breakdown of cases. The help of native language speakers was enlisted for translations where required. Moreover, for any countries where these data were difficult to find, the national agencies were contacted to obtain any publicly available datasets, reports or data summaries. Despite efforts, data were unavailable for some countries and years, while in some cases only high level data summaries were publicly available, lacking sufficient geographical disaggregation for inclusion in the analyses presented in the main paper. Table S1 documents the national agency report sources used per-country, while figure S1 plots the years of data covered by the reports, figure S2 maps the countries covered for each time period, and supplemental file 1 provides the full range of summary numbers extracted from the reports.

Academic papers

The data compiled from national agency reports were complemented by web searches on PubMed, Web of Science, Google Scholar and standard Google search for 'imported malaria' and the name of the country in question, in both English and the primary language of the country where this was not English. These searches identified a set of additional academic papers and reports documenting summaries of imported cases. Those papers that reported imported malaria cases from a specific hospital, facility or subnational region of a country were discarded, so that only national-level numbers remained. For each country, where available, data were extracted on the numbers of confirmed cases reported, the year, their likely origin regions and/or countries, the species of parasite and the method of diagnosis. Often these papers documented and analysed the same data that were covered by national agency reports, and therefore, comparisons were initially made to ensure that double-counting of cases did not occur. Where duplication was evident through numbers in academic papers being obtained from national reporting systems, the national agency report statistics were prioritised over the academic paper summaries. Data were unavailable for some countries and years in both academic publications, while in some cases only high level data summaries were published in the academic papers, lacking sufficient geographical disaggregation for inclusion in the analyses presented in the main paper. Table S1 documents the academic paper sources used per-country, while figure S1 plots the years of data covered by them, figure S2 maps the countries covered for each time period, and supplemental file 1 provides summary numbers extracted from the reports.

Country	References
Albania	1
Australia	2*,3*,4*,5*,6*
Austria	7,8,9,10

Bahrain	11
Belgium	12,13,14
Bulgaria	15
Canada	16,17
Caribbean	18
Chile	19
Croatia	20,21
Czech Republic	22
Denmark	23*,24,25*,26*,27*,28*
Estonia	29,30*,31*,32*,33*
Europe	34
Finland	35,36,37,38,39*,40*,41*,42*
France	43,44,45,46,47,48,49,50,51,52,53,54,55,56,57,58,59*,60,61,62,63*,64*,65*,66*,67*,68,69*
Germany	70,71,72,73,74,75*,76,77*,78*,79*,80*,81*,82
Greece	83,84*,85,86,87
Hong Kong	88*
Hungary	89,90*,91*,92*
Ireland	93*,94*,95*,96*,97*,98*,99,100
Israel	101,102
Italy	103,104,105,106,107,108,109,110,111,112,113,114,115,116,117,118,119,120,121
Jamaica	122
Japan	123,124,125
Lithuania	126,127,128
Mayotte	44
Morocco	129*
Netherlands	130,131,132,133,134,14

New Zealand	135*,136*,137*,138*,139*,140*,141*,142*,143*,144*
Northern Ireland	145,146
Norway	147,148,149*,150,151,152
Poland	153,154*,155*,156*,157*,158*,159*
Portugal	160,161,162,163,164,165,166*,167
Qatar	168,169*
Réunion Island	170,44
Romania	171,172,173,174,175
Saudi Arabia	176
Scotland	177
Serbia	178,179*,180*
Singapore	181
Slovak Republic	182,183,184
Slovenia	185*,186
Spain	187,188,189,190,191,192*,193,194,195,196,197,198,199,200,201*,202*
Sweden	203,204*,205*
Switzerland	206,207,208,209,210,211*,212,213*
Taiwan	214*
Trinidad	215
United Kingdom	216,217,218,219*,220,217,221,222,223,224,177,225*,226,227,228*,229*,230*,231*,100,232*,233*,234*,235*,236*,237*,238*,239*
United States	240,241*,102,242*,243*,244*,245*,246*,247*,248*,249*,250*,251*,252*,253*,254*,255*,256*

* Official statistics

Table S1. The publicly available data sources used for each country in the analyses presented.

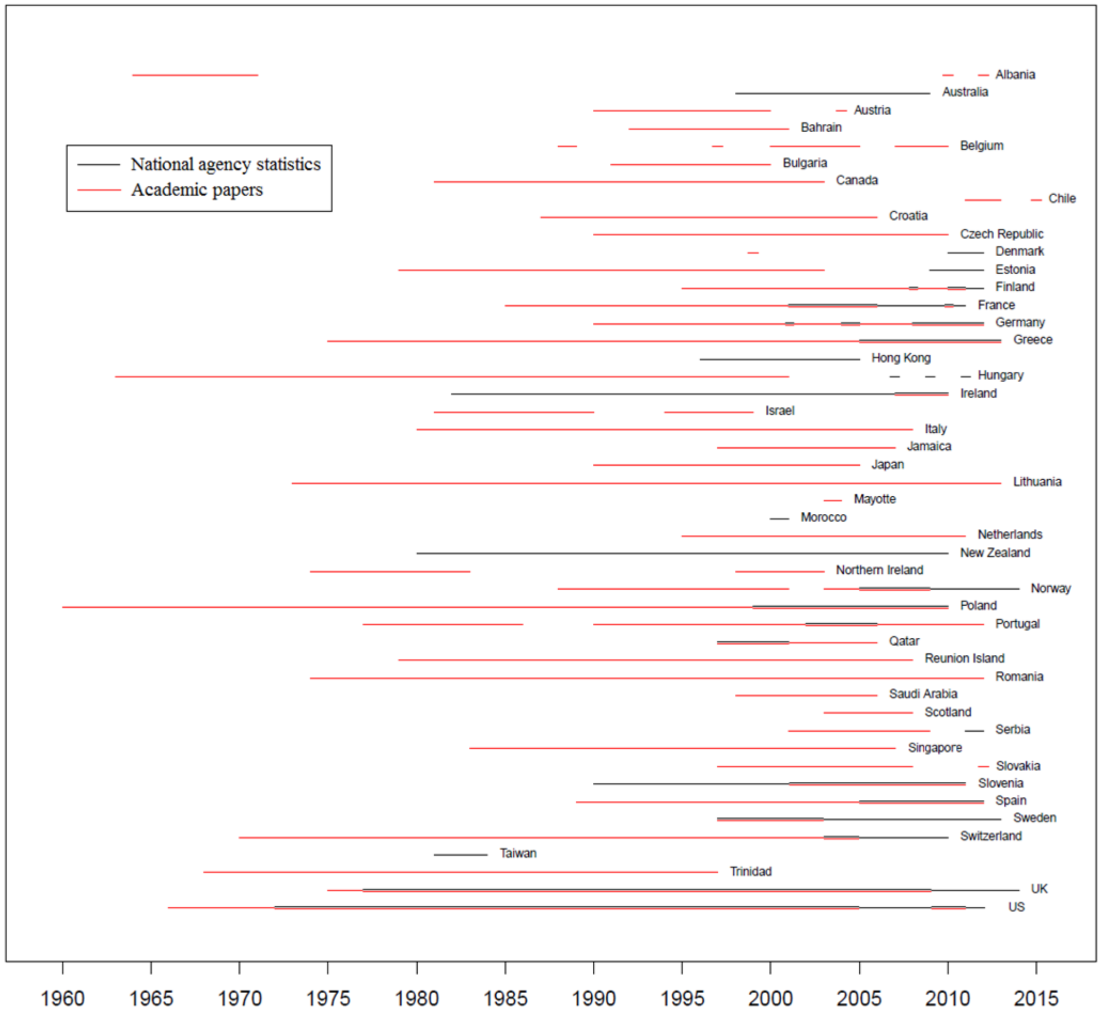

Figure S2. Datasets assembled for each non-endemic malaria country. Sources and data extractions are provided in table S1 and supplemental file 1.

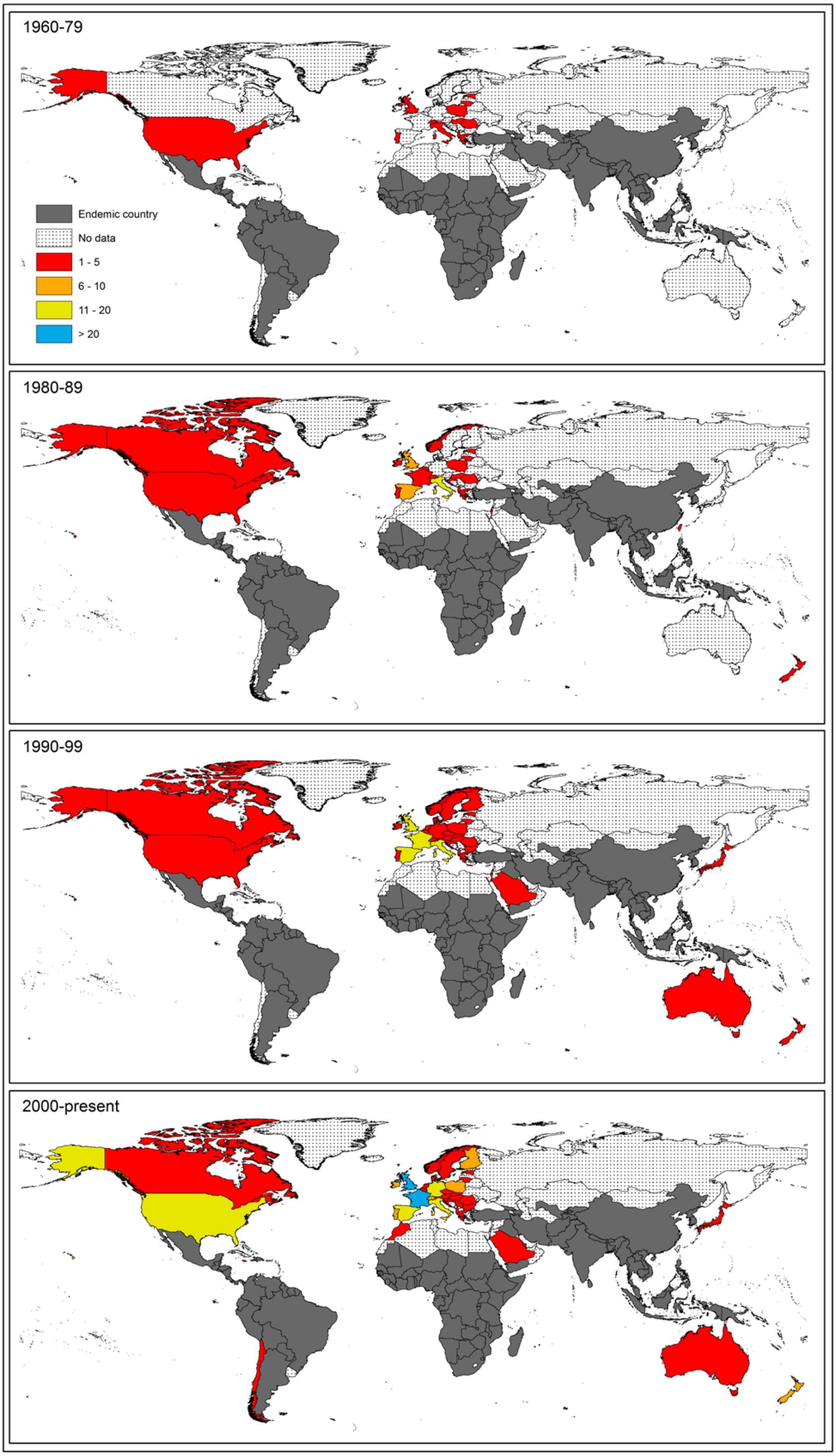
1960-79
Endemic country
No data
1 - 5
6 - 10
11 - 20
> 20
1980-89
1990-99
2000-present

Figure S3. Maps showing assignment of endemic countries and numbers of national agency reports and academic papers from which imported malaria case statistics were extracted for each country across four time periods covering 1960-2015.

Data processing

The reports, papers and resulting datasets assembled covered a wide range of levels of detail, temporal coverage, diagnostic capabilities and sample sizes (Table S1, figures S2-4, supplemental file 1). In the majority of cases, the data required transcribing from PDF documents. To reduce the possibility of error in undertaking this, the initial transcription undertaken was checked independently by a second researcher.

To ensure a degree of comparability across countries, a set of broad rules were constructed to facilitate data summarization, exclusion and processing. These were based on achieving a balance between maintaining a wide representation of data from multiple countries, time periods and sources, and implementing some quality control to ensure comparability between datasets and avoid double-counting. Different hierarchies of analysis were undertaken to enable presentations of outputs where (i) data inclusion criteria were relatively relaxed to enable comparisons across many countries, and (ii) criteria were tightened or data were aggregated to facilitate the production of more robust, but less detailed, conclusions. The data were processed to obtain information on specific features of imported malaria geography, namely origins, destinations, numbers of cases, species type and diagnostic capabilities (in non-endemic countries). Supplemental file 1 presents the data extracted under these different headings.

Though the datasets assembled extended from 1960 to the present day (figure S1), to obtain a contemporary picture while still including a large number of countries/regions, analyses were undertaken only on the most recent ten years of data. The data analyses were therefore restricted to the 2005-15 period, using an annual mean of cases across the full ten-year period where available, though for some countries, data were only available for less than five years of this time period. In a few cases where 2005-2015 data were lacking for a country (table S1, figure S1), 2000-05 data were considered. For each endemic ‘exporting’ country, all reported annual mean case numbers exported to non-endemic reporting countries were aggregated to obtain estimates of the proportions of each parasite species (or mixed infections, where documented as such) exported (figure 4, table 1 in main paper). While this averaging masked temporal trends existing in the data, for most countries clear trends over time were not apparent, with species compositions generally remaining stable across the period. Moreover, given the gaps existing in publicly available data (table S1), this time window facilitated the inclusion of many more countries than a more constrained one.

The data were processed to obtain origin-destination matrices for average numbers of cases per year imported from endemic to non-endemic countries (figure 1 in main paper). Many data sources reported the sources of cases only by large regions, therefore a regional version of this matrix was also constructed to enable the inclusion of more data and thus identify geographical patterns more robustly. The data were also analysed to obtain estimates of the aggregate malaria species compositions being exported from endemic countries and imported to non-endemic countries. Where species breakdowns of imported cases were reported, these were aggregated and summarized across the reporting period. Similarly to the origin-destination matrices, for many countries data on species composition were only reported by origin region, thus data were also aggregated by region to provide larger sample sizes and thus more confidence in estimates of differences between regions by composition (Figure 3 in the main paper).

Diagnostic capacities

To provide context to the datasets assembled and analyses undertaken, where available, information of diagnostic methods used to obtain the imported malaria data presented were gathered. The large percentages of 'unknown' malaria types for some countries (Figures 3 and 4, table 1 in main paper) are likely to be indicative of a lack of diagnostic capacity, while differences in diagnostic methods may mean that some data are more reliable in terms of their speciation than others. Figure S4 presents a summary of available data on diagnostic approaches used in European countries for the imported malaria datasets analysed, and highlights the mixed picture that exists. Those countries that receive higher numbers of imported malaria cases (e.g. France, Italy, UK, Germany, Spain) generally use PCR more often than the smaller countries that typically receive lower numbers of cases, and rely on microscopy for malaria confirmation and speciation.

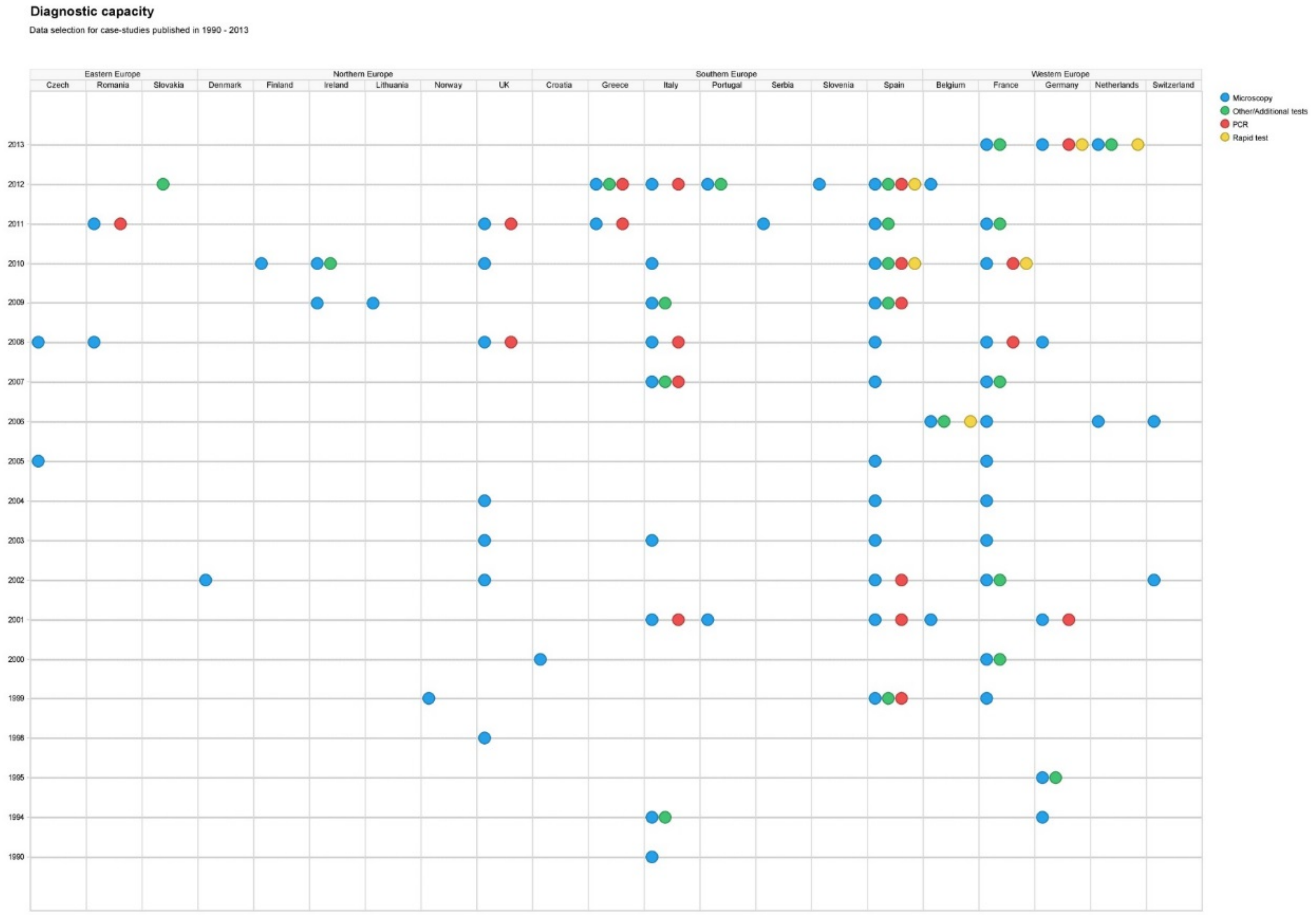

Figure S4. Methods used for diagnosis of imported malaria cases in European countries 1990-2013, where reported, in the publicly available data sources used in the analyses presented.

Network community detection

Communities in a network reflect a partition of nodes that are densely connected and separated from the other nodes in the network, and thus they share common properties and play similar roles within the graph. By mapping communities on the imported malaria network defined here, we aimed to identify groups of countries that show strong links in terms of movements of infected travellers. Newman and Girvan [257] define a modularity score, which measures the quality of network partitions as:

$$Q = \frac{1}{2m}\sum_{i,j}[W_{ij} - \frac{k_i k_j}{2m}]\delta(c_i, c_j)$$

In which, W_{ij} represents the weight of the edge between country *i* and country *j* (here this is the number of cases moving from *i* to *j*), $k_i = \Sigma_j W_{ij}$ is the sum of the weights of the connections attached to country *i*, c_i is the community to which country *i* is assigned, $\sigma(u,v)$ is 1 if $u = v$, otherwise $m = \frac{1}{2} \Sigma W_{ij}$. The analyses utilized a multilevel algorithm for community detection.[258] This method utilizes an iterative approach that merges communities to maximize the modularity score: Firstly modularity was optimized by allowing only local changes of communities, secondly the established communities were combined together to construct a new network. These two passes were repeated iteratively until no increase of modularity was possible. The number of communities returned by this algorithm yields the maximum modularity score.

References

1 Bino, S. in *ProMed Newsgroup* (2012).

2 Liu, C., Broom, A. K., Kurucz, N. & Whelan, P. I. Communicable Diseases Network Australia: National Arbovirus and Malaria Advisory Committee annual report 2004-05. *Commun Dis Intell* **29**, 341-357 (2005).

3 Liu, C., Johansen, C., Kurucz, N. & Whelan, P. Communicable Diseases Network Australia National Arbovirus and Malaria Advisory Committee annual report, 2005-06. *Commun Dis Intell* **30**, 411-429 (2006).

4 Liu, C. *et al.* Communicable Diseases Network Australia National Arbovirus and Malaria Advisory Committee annual report, 2006-07. *Commun Dis Intell* **32**, 31-47 (2008).

5 Fitzsimmons, G. J., Wright, P., Johansen, C. A. & Whelan, P. I. Arboviral diseases and malaria in Australia, 2007/08: annual report of the National Arbovirus and Malaria Advisory Committee. *Commun Dis Intell* **33**, 155-169 (2009).

6 Fitzsimmons, G. J., Wright, P., Johansen, C. A. & Whelan, P. I. Arboviral diseases and malaria in Australia, 2008-09: annual report of the National Arbovirus and Malaria Advisory Committee. *Commun Dis Intell* **34**, 225-240 (2010).

7 Strauss, R. & Pfeifer, C. Malaria in Austria 1990-2000. *Eurosurveillance* **8**, 91-96, doi:408 [pii] (2003).

8 Twisselmann, B. Malaria in Austria, 1990-9. *Eurosurveillance Weekly Release* **5**, 1832 (2001).

9 Buchstaller, G. & Dieberger, H. Importierte Malaria in Wien 1996-2000. *Mitt Osterr Ges Tropenmed Parasitol* **23**, 63-66 (2001).

10 El Belazi, G. & Klein, J. P. Malaria in Österreich im Jahr 2004. (2005).

11 Ismaeel, A. Y., Senok, A. C., Al-Khaja, K. A. J. & Botta, G. A. Status of Malaria in the Kingdom of Bahrain: a 10-year Review. *Journal of travel medicine* **11**, 97-101 (2004).

12 Van den Ende, J. *et al.* Changing epidemiological and clinical aspects of imported malaria in Belgium. *Journal of travel medicine* **8**, 19-25 (2001).

13 Bottieau, E. *et al.* Imported non-Plasmodium falciparum malaria: a five-year prospective study in a European referral center. *Am J Trop Med Hyg* **75**, 133-138, doi:75/1/133 [pii] (2006).
14 Kreeftmeijer-Vegter, A. R. *et al.* Treatment outcome of intravenous artesunate in patients with severe malaria in the Netherlands and Belgium. *Malar J* **11**, 102, doi:10.1186/1475-2875-11-102 (2012).
15 Kurdova, R. I., Vutchev, D. I. & Petrov, P. P. Malaria situation in Bulgaria and surveillance measures (1991-2000). *Global NEST: The International Journal* **3**, 153-162 (2001).
16 MacLean, J. D. *et al.* Malaria Epidemics and Surveillance Systems in Canada. *Emerging infectious diseases* **10**, 1197-1201 (2004).
17 Boggild, A. K., Yohanna, S., Keystone, J. S. & Kain, K. C. Prospective Analysis of Parasitic Infections in Canadian Travelers and Immigrants. *Journal of travel medicine* **13**, 138-144, doi:10.1111/j.1708-8305.2006.00032.x (2006).
18 Rawlins, S. C., Hinds, A. & Rawlins, J. M. Malaria and its Vectors in the Caribbean: The Continuing Challenge of the Disease Forty-Five Years after Eradication from the Islands. *West Indian Med J* **57**, 462-469 (2008).
19 Weitzel, T. *et al.* Cluster of Imported Vivax Malaria in Travelers Returning From Peru. *Journal of travel medicine*, doi:10.1111/jtm.12234 (2015).
20 Peric, D., Skrobonja, I. & Skrobonja, A. [Malaria in Croatia in the period between 1987 to 2006]. *Lijec Vjesn* **131**, 192-195 (2009).
21 Nikolić, N., Poljak, I. & Trošelj-Vukić, B. Malaria, a travel health problem in the maritime community. *Journal of travel medicine* **7**, 309-314 (2000).
22 Stejskal, F. & Trojanek, M. Tropical and Travel Medicine in the Czech Republic. (Department of Tropical Medicine, Prague, 2010).
23 Department of Epidemiology. NATIONAL SURVEILLANCE OF COMMUNICABLE DISEASES. 1-2 (Statens Serum Institut, Copenhagen, Denmark, 2008).
24 Twisselmann, B. Malaria in Denmark, 1999. *Eurosurveillance Weekly Release* **4**, 1526 (2000).
25 Department of Epidemiology. NATIONAL SURVEILLANCE OF COMMUNICABLE DISEASES. 1-2 (Statens Serum Institut, Copenhagen, Denmark, 2009).
26 Department of Epidemiology. NATIONAL SURVEILLANCE OF COMMUNICABLE DISEASES. 1-2 (Statens Serum Institut, Copenhagen, Denmark, 2010).
27 Department of Epidemiology. NATIONAL SURVEILLANCE OF COMMUNICABLE DISEASES. 1-2 (Statens Serum Institut, Copenhagen, Denmark, 2011).
28 Department of Epidemiology. Malaria 2012. (Statens Serum Institut, Denmark, 2013).
29 Epštein, J. & Järvelaid, M. Epidemioloogiline ülevaade malaariasse haigestumisest. *Eesti Arst* **84**, 25-30 (2005).
30 Terviseamet Health Board. Travel-related infectious diseases in the 2009. (2010).
31 Terviseamet Health Board. Travel-related infectious diseases in the 2010. (2011).
32 Terviseamet Health Board. Travel-related infectious diseases in the 2011. (2012).
33 Terviseamet Health Board. Travel-related infectious diseases in the 2012. (2013).
34 Behrens, R. H. *et al.* Malaria prophylaxis policy for travellers from Europe to the Indian Sub Continent. *Malaria Journal* **5**, 7, doi:10.1186/1475-2875-5-7 (2006).
35 Guedes, S., Siikamäki, H., Kantele, A. & Lyytikäinen, O. Imported Malaria in Finland 1995 to 2008: An Overview of Surveillance, Travel Trends, and Antimalarial Drug Sales. *Journal of travel medicine* **17**, 400-404, doi:10.1111/j.1708-8305.2010.00456.x (2010).
36 Siikamäki, H. Imported malaria in Finland, 2001. *Eurosurveillance Weekly Release* **6**, 2126 (2002).
37 Siikamäki, H., Kivelä, P., Lyytikäinen, O. & Kantele, A. Imported malaria in Finland 2003–2011: prospective nationwide data with rechecked background information. *Malaria Journal* **12** (2013).

38 Valve, K., Ruotsalainen, E., Karki, T., Pekkanen, E. & Siikamaki, H. Cluster of imported malaria from Gambia in Finland--travellers do not listen to given advice. *Eurosurveillance* **13** (2008).
39 National Institute for Health and Welfare. Infectious Diseases in Finland 2008. (2009).
40 National Institute for Health and Welfare. Infectious Diseases in Finland 2010. (2011).
41 National Institute for Health and Welfare. Infectious Diseases in Finland 2011. (2012).
42 National Institute for Health and Welfare. Infectious Diseases in Finland 2012. (2013).
43 Legros, F., Gay, F., Belkaid, M. & Danis, M. Imported malaria in continental France in 1996. *Eurosurveillance* **3**, 37-38, doi:105 [pii] (1998).
44 Legros, F., Arnaud, A., El Mimouni, B. & Danis, M. Paludisme d'importation en France métropolitaine: données épidémiologiques 2001-2004. *Bulletin épidémiologique hebdomadaire* **32** (2006).
45 Bruneel, F. *et al.* The clinical spectrum of severe imported falciparum malaria in the intensive care unit: report of 188 cases in adults. *Am J Respir Crit Care Med* **167**, 684-689, doi:10.1164/rccm.200206-631OC (2003).
46 Briand, V. *et al.* Hospitalization criteria in imported falciparum malaria. *Journal of travel medicine* **14**, 306-311, doi:10.1111/j.1708-8305.2007.00143.x (2007).
47 Malvy, D. *et al.* Risk of malaria among French adult travellers. *Travel Med Infect Dis* **4**, 259-269, doi:10.1016/j.tmaid.2005.07.002 (2006).
48 Castela, F., Legros, F. & Lagardere, B. [Imported malaria in children in France]. *Arch Pediatr* **10**, 758-765 (2003).
49 Legros, F. *et al.* Risk Factors for Imported Fatal *Plasmodium falciparum* Malaria, France, 1996–2003. *Emerg Infect Dis.*, 1-14 (2007).
50 Seringe, E. Severe Imported Plasmodium falciparum Malaria, France, 1996–2003. *Emerging infectious diseases*, doi:10.3201/eid1705.101527 (2011).
51 Badiaga, S. *et al.* Severe imported malaria: clinical presentation at the time of hospital admission and outcome in 42 cases diagnosed from 1996 to 2002. *J Emerg Med* **29**, 375-382, doi:10.1016/j.jemermed.2005.03.009 (2005).
52 Mouala, C. *et al.* Imported malaria in HIV-infected patients enrolled in the ANRS CO4 FHDH study. *Journal of acquired immune deficiency syndromes* **49**, 55-60, doi:10.1097/QAI.0b013e31817e635b (2008).
53 Eloy, O. *et al.* Paludisme d’importation de l’enfant. Expérience du centre hospitalier de Versailles (1997-2001). *Annales de Biologie Clinique* **61**, 449-453 (2003).
54 Corne, P. *et al.* Paludisme grave d'importation chez l'adulte: étude rétrospective de 32 cas admis en réanimation. *Pathologie Biologie* **52**, 622-626 (2004).
55 Ranque, S. *et al.* Treatment of imported malaria in adults: a multicentre study in France. *QJM* **98**, 737-743, doi:10.1093/qjmed/hci110 (2005).
56 Dubos, F. *et al.* Imported malaria in children: incidence and risk factors for severity. *Diagn Microbiol Infect Dis* **66**, 169-174, doi:10.1016/j.diagmicrobio.2009.08.018 (2010).
57 Pistone, T. *et al.* [Imported malaria in University Hospital Center of Bordeaux, France, 2000-2007. A comparison study with the French national epidemiological data]. *Bulletin de la Societe de pathologie exotique* **103**, 104-110, doi:10.1007/s13149-010-0045-4 (2010).
58 Parola, P. *et al.* [Imported malaria at the Marseilles Hopital-Nord, France: a prospective study on 352 cases between 2001 and 2003]. *Med Mal Infect* **35**, 482-488, doi:10.1016/j.medmal.2005.05.006 (2005).
59 National Reference Centre for Malaria metropolitan France. Activity Report 2001-2005 CRN Malaria (INVS, 2006).
60 Chalumeau, M. *et al.* Delay in diagnosis of imported Plasmodium falciparum malaria in children. *European journal of clinical microbiology & infectious diseases : official publication of the European Society of Clinical Microbiology* **25**, 186-189, doi:10.1007/s10096-006-0105-3 (2006).

61 Ansart, S. *et al.* Predictive factors of imported malaria in 272 febrile returning travelers seen as outpatients. *Journal of travel medicine* **17**, 124-129, doi:10.1111/j.1708-8305.2009.00382.x (2010).
62 Pillot Debelleix, M. *et al.* [One hundred and fifteen cases of imported falciparum malaria admitted at the Bordeaux teaching hospital Emergency Unit]. *Med Mal Infect* **40**, 88-93, doi:10.1016/j.medmal.2009.08.008 (2010).
63 National Reference Centre for Malaria metropolitan France. Activity Report 2006 CRN Malaria (INVS, 2007).
64 National Reference Centre for Malaria metropolitan France. Activity Report 2007 CNR Malaria (INVS, 2008).
65 National Reference Centre for Malaria metropolitan France. Activity Report 2008 CNR Malaria (INVS, 2009).
66 National Reference Centre for Malaria metropolitan France. Activity Report 2009 CRN Malaria (INVS, 2010).
67 National Reference Centre for Malaria metropolitan France. Activity Report 2010 CNR Malaria (INVS, 2011).
68 Rapp, P. C. (ed Hôpital Bégin Service des maladies infectieuses et tropicales, Saint-Mandé) (2012).
69 National Reference Centre for Malaria metropolitan France. Activity Report 2011 CNR Malaria (INVS, 2012).
70 Jelinek, T., Nothdurft, H. D. & Loscher, T. Malaria in Nonimmune Travelers: A Synopsis of History, Symptoms, and Treatment in 160 Patients. *Journal of travel medicine* **1**, 199-202 (1994).
71 Apitzsch, L., Rasch, G. & Kiehl, W. Imported malaria in Germany in 1996. *Eurosurveillance* **3**, 35-36, doi:104 [pii] (1998).
72 Schoneberg, I., Stark, K., Altmann, D. & Krause, G. [Imported malaria in Germany--countries of infection and parasite species, 1993-2007]. *Gesundheitswesen* **70**, 256-261, doi:10.1055/s-2008-1077056 (2008).
73 Schwake, L. *et al.* Early treatment of imported falciparum malaria in the intermediate and intensive care unit setting: an 8-year single-center retrospective study. *Crit Care* **12**, R22, doi:10.1186/cc6796 (2008).
74 Sabatinelli, G., Ejov, M. & Joergensen, P. Malaria in the WHO European Region (1971-1999). *Eurosurveillance* **6**, 61-65, doi:213 [pii] (2001).
75 Robert Koch-Institut. Travel Associated Infectious Diseases in 2001. *Epidemiological Bulletin* (2002).
76 Flüß, F., Lenhartz, H. & Höger, P. H. Malaria bei Kindern in Deutschland. *Monatsschrift Kinderheilkunde* **161**, 530-534 (2013).
77 Robert Koch-Institut. Travel Associated Infectious Diseases in 2004-2005. *Epidemiological Bulletin* (2006).
78 Robert Koch-Institut. Travel Associated Infectious Diseases in 2008. *Epidemiological Bulletin* (2009).
79 Robert Koch-Institut. Travel Associated Infectious Diseases in 2009. *Epidemiological Bulletin* **38** (2010).
80 Robert Koch-Institut. Travel Associated Infectious Diseases in 2010. *Epidemiological Bulletin* **41** (2011).
81 Robert Koch-Institut. Travel Associated Infectious Diseases in 2011-2012. *Epidemiological Bulletin* (2013).
82 Stark, K. & Schöneberg, I. Increase in malaria cases imported from Pakistan to Germany in 2012. *Eurosurveillance* **17** (2012).
83 Vakali, A. *et al.* Malaria in Greece, 1975 to 2010. *Eurosurveillance* **17** (2012).
84 Hellenic Center for Disease Control and Prevention. Malaria in Greece 2005-2013. (2013).

85 Pavli, A. *et al.* Descriptive analysis of malaria prophylaxis for travellers from Greece visiting malaria-endemic countries. *Travel Med Infect Dis* **9**, 284-288, doi:10.1016/j.tmaid.2011.09.005 (2011).
86 Danis, K. *et al.* Autochthonous Plasmodium vivax malaria in Greece, 2011. *Eurosurveillance* **16** (2011).
87 Ejov, M., Domanović, D., Zeller, H. & Van Bortel, W. Joint WHO–ECDC mission related to local malaria transmission in Greece, 2012. (2012).
88 Centre for Health Protection. Epidemiology of Malaria in Hong Kong. (2006).
89 JOHAN BÉLA National Centre for Epidemiology. The import of malaria in Hungary: current situation and protection. *Weekly Epidemiological Information* **9** (2002).
90 National Center for Epidemiology. Epidemiological Information 2007. (2008).
91 National Center for Epidemiology. Epidemiological Information 2009. (2010).
92 National Center for Epidemiology. Epidemiological Information 2011. (2012).
93 HPSC Vectorborne Disease Sub-Committee. Health Protection Surveillance Centre Annual Report 2005. (2006).
94 HPSC Vectorborne Disease Sub-Committee. Health Protection Surveillance Centre Annual Report 2006. (2007).
95 HPSC Vectorborne Disease Sub-Committee. Health Protection Surveillance Centre Annual Report 2007. (2008).
96 HPSC Vectorborne Disease Sub-Committee. Health Protection Surveillance Centre Annual Report 2008. (2009).
97 HPSC Vectorborne Disease Sub-Committee. Health Protection Surveillance Centre Annual Report 2009. (2010).
98 HPSC Vectorborne Disease Sub-Committee. Health Protection Surveillance Centre Annual Report 2010. (2011).
99 Leahy, T. R., Malikiwi, A., Cafferkey, M. & Butler, K. M. Imported childhood malaria: the Dublin experience, 1999-2006. *Ir J Med Sci* **178**, 329-332, doi:10.1007/s11845-009-0343-3 (2009).
100 Ladhani, S. *et al.* Prospective, national clinical and epidemiologic study on imported childhood malaria in the United Kingdom and the Republic of Ireland. *Pediatr Infect Dis J* **29**, 434-438, doi:10.1097/INF.0b013e3181c4d97c (2010).
101 Dan, M., Costin, C. & Slater, P. E. Malaria Imported by Travelers: The Israeli Experience. *Journal of travel medicine* **3**, 182-185 (1996).
102 Schwartz, E., Parise, M., Kozarsky, P. & Cetron, M. Delayed onset of malaria--implications for chemoprophylaxis in travelers. *The New England journal of medicine* **349**, 1510-1516, doi:10.1056/NEJMoa021592349/16/1510 [pii] (2003).
103 Majori, G., Sabatinelli, G., Casaglia, O., Cavallini, C. & Monzali, C. Imported malaria in Italy from 1986 to 1988. *Journal of the Royal Society of Health* **110**, 88-89 (1990).
104 Calleri, G., Macor, A., Leo, G. & Caramello, P. Imported Malaria in Italy: Epidemiologic and Clinical Studies. *Journal of travel medicine* **1**, 231-234 (1994).
105 Raglio, A. *et al.* Ten-year Experience with Imported Malaria in Bergamo, Italy. *Journal of travel medicine* **1**, 152-155 (1994).
106 Spinazzola, F. *et al.* Imported malaria at Italy's National Institute for Infectious Diseases Lazzaro Spallanzani, 1984-2003. *European journal of clinical microbiology & infectious diseases : official publication of the European Society of Clinical Microbiology* **26**, 175-179, doi:10.1007/s10096-007-0266-8 (2007).
107 Sabatinelli, G. & Majori, G. Malaria surveillance in Italy : 1986-1996 analysis and 1997 provisional data. *Euro Surveill* **3**, 38-40 (1998).
108 Foca, A. *et al.* Fourteen-year experience with imported malaria. *Infez Med* **12**, 186-192 (2004).

109 Di Perri, G. *et al.* West African Immigrants and New Patterns of Malaria Imported to North Eastern Italy. *Journal of travel medicine* **1**, 147-151 (1994).
110 Romi, R., Sabatinelli, G. & Majori, G. Malaria epidemiological situation in Italy and evaluation of malaria incidence in Italian travelers. *Journal of travel medicine* **8**, 6-11 (2001).
111 Romi, R., Boccolini, D. & Majori, G. Malaria incidence and mortality in Italy in 1999-2000. *Eurosurveillance* **6**, 143-147 (2001).
112 Mascarello, M. *et al.* Imported malaria in immigrants to Italy: a changing pattern observed in north eastern Italy. *Journal of travel medicine* **16**, 317-321, doi:10.1111/j.1708-8305.2009.00321.x (2009).
113 Matteelli, A., Colombini, P., Gulletta, M., Castelli, F. & Carosi, G. Epidemiological features and case management practices of imported malaria in northern Italy 1991-1995. *Tropical medicine & international health : TM & IH* **4**, 653-657 (1999).
114 Nicastri, E. *et al.* Plasmodium falciparum multiple infections, disease severity and host characteristics in malaria affected travellers returning from Africa. *Travel Med Infect Dis* **6**, 205-209, doi:10.1016/j.tmaid.2008.01.001 (2008).
115 Paglia, M. G. *et al.* Molecular diagnosis and species identification of imported malaria in returning travellers in Italy. *Diagn Microbiol Infect Dis* **72**, 175-180, doi:10.1016/j.diagmicrobio.2011.09.013 (2012).
116 Romi, R. *et al.* Incidence of malaria and risk factors in Italian travelers to malaria endemic countries. *Travel Medicine and Infectious Disease* **8**, 144-154, doi:10.1016/j.tmaid.2010.02.001 (2010).
117 Romi, R. *et al.* Malaria Surveillance in Italy: The 2000-2008 National Pattern of Imported Cases. *Italian Journal of Tropical Medicine* **15**, 35-37 (2010).
118 Calderaro, A. *et al.* An 8-year survey on the occurrence of imported malaria in a nonendemic area by microscopy and molecular assays. *Diagn Microbiol Infect Dis* **61**, 434-439, doi:10.1016/j.diagmicrobio.2008.03.016 (2008).
119 Mascarello, M. *et al.* Imported Malaria in Adults and Children: Epidemiological and Clinical Characteristics of 380 Consecutive Cases Observed in Verona, Italy. *Journal of travel medicine* **15**, 229-236, doi:10.1111/j.1708-8305.2008.00204.x (2008).
120 Boccolini, D., Romi, R., D' Amato, S., Pompa, M. G. & Majori, G. Malaria Surveillance in Italy and analysis of the case reports 2002-2006. *Giornale Italiano Di Medicina Tropicale* **12** (2007).
121 Peruzzi, S. *et al.* Prevalence of imported malaria in Parma during 2005-2006. *Acta Biomed* **78**, 170-175 (2007).
122 Lewis-Fuller, E. (Ministry of Health, Jamaica, 2008).
123 Kimura, M. *et al.* Epidemiological and Clinical Aspects of Malaria in Japan. *Journal of travel medicine* **10**, 122-127 (2003).
124 Kano, S. & Kimura, M. Trends in malaria cases in Japan. *Acta Tropica* **89**, 271-278, doi:10.1016/j.actatropica.2003.10.003 (2004).
125 Tada, Y., Okabe, N. & Kimura, M. Travelers' risk of malaria by destination country: A study from Japan. *Travel Medicine and Infectious Disease* **6**, 368-372, doi:10.1016/j.tmaid.2008.08.002 (2008).
126 Scerbaviciene, R. & Pilipavicius, R. Malaria among seamen in Klaipeda during 1973-1998. *Int Marit Health* **50**, 7-13 (1999).
127 Scerbaviciene, R. & Pilipavicius, R. Malaria among seamen in Klaipeda in 1999-2008. *Int Marit Health* **60**, 29-32 (2009).
128 Communicable Diseases and AIDS Centre (ULAC). World Malaria Day: Vaccines are not to prevent the disease helps the regular medicines. (2013).
129 National Institute of Hygiene. Report on malaria situation in Morocco during 2001. (2002).
130 Bosman, A. & Chaves, S. Overview of malaria notifications in the Netherlands, 1995-9. *Eurosurveillance* **4**, 1527 (2000).

131 Vliegenthart-Jongbloed, K. *et al.* Severity of imported malaria: protective effect of taking malaria chemoprophylaxis. *Malar J* **12**, 265, doi:10.1186/1475-2875-12-265 (2013).
132 Van Rijckevorsel, G. *et al.* Incidence and trends of imported malaria in the Netherlands: 2000-2007. *International Journal of Infectious Diseases* **14**, e25, doi:10.1016/j.ijid.2010.02.1543 (2010).
133 Baas, M. C., Wetsteyn, J. C. & van Gool, T. Patterns of imported malaria at the academic medical center, Amsterdam, the Netherlands. *Journal of travel medicine* **13**, 2-7, doi:10.1111/j.1708-8305.2006.00003.x (2006).
134 Driessen, G. J., Pereira, R. R., Brabin, B. J. & Hartwig, N. G. Imported malaria in children: a national surveillance in the Netherlands and a review of European studies. *The European Journal of Public Health* **18**, 184-188, doi:10.1093/eurpub/ckm101 (2007).
135 Sneyd, E., Lopez, L., Eglinton, M., McDowell, R. & Margolin, T. Infectious Diseases in New Zealand: 2001 Annual Surveillance Summary. 1-106 (Institute of Environmental Science and Research, Wellington 2002).
136 Sneyd, E. & Baker, M. Infectious Diseases in New Zealand: 2002 Annual Surveillance Summary. 1-179 (Institute of Environmental Science and Research, Wellington, 2003).
137 Population and Environmental Health Group. Notifiable and other diseases in New Zealand annual report 2003. 1-71 (Institute of Environmental Science and Research, Wellington 2004).
138 Population and Environmental Health Group. Notifiable and other diseases in New Zealand annual report 2004. 1-72 (Institute of Environmental Science and Research, Wellington, 2005).
139 Population and Environmental Health Group. Notifiable and other diseases in New Zealand annual report 2005. 1-66 (Institute of Environmental Science and Research, Wellington, 2006).
140 Population and Environmental Health Group. Notifiable and other diseases in New Zealand annual report 2006. 1-70 (Institute of Environmental Science and Research, Wellington, 2007).
141 Population and Environmental Health Group. Notifiable and other diseases in New Zealand annual report 2007. 1-68 (Institute of Environmental Science and Research, Wellington, 2008).
142 Population and Environmental Health Group. Notifiable and other diseases in New Zealand annual report 2008. 1-67 (Institute of Environmental Science and Research, Wellington, 2009).
143 Population and Environmental Health Group. Notifiable and other diseases in New Zealand annual report 2009. 1-72 (Institute of Environmental Science and Research, Wellington, 2010).
144 The Health Intelligence Team. Notifiable and other diseases in New Zealand Annual Report 2010. 1-114 (Institute of Environmental Science and Research, Wellington, 2011).
145 Gillespie, S. H. & Canavan, D. A. Malaria in Northern Ireland. *Ulster Med J* **55**, 57-60 (1986).
146 Ong, G. M. & Smyth, B. Imported malaria to Northern Ireland: improving surveillance for better intervention. *Ulster Med J* **75**, 129-135 (2006).
147 Jensenius, M. *et al.* Low frequency of complications in imported falciparum malaria: a review of 222 cases in south-eastern Norway. *Scand J Infect Dis* **31**, 73-78 (1999).
148 Blystad, H. & Løvoll, Ø. Imported malaria in Norway. *Eurosurveillance* **6**, 2115 (2002).
149 Norwegian Institute of Public Health. Surveillance of Communicable Diseases and Nosocomial Infections in Norway 2006. 1-52 (Department of Infectious Disease Epidemiology, Oslo, 2007).
150 Sandbu, S. Prevention of malaria in travelers. (Division of Infectious Disease, Institute of Public Health, Norway, 2009).

151 National Institute of Public Health. in *Smittevernveilederen (Smittevernboka)* (ed Hans Blystad) (2015).
152 Mørch, K. *et al.* Severe malaria and artesunate treatment, Norway. *Emerging infectious diseases* **14**, 1816-1818, doi:10.3201/eid1411.080636 (2008).
153 Stępień, M. The epidemiological situation of malaria in Poland. (2012).
154 National Institute of Hygiene. (Poland, 2011).
155 Rosińska, M. Malaria in Poland in 2005. *Przegl Epidemiol* **61**, 323-324 (2007).
156 Rosińska, M. Malaria in Poland in 2007. (Department of Epidemiology, National Institute of Public Health - National Institute of Hygiene, Warsaw, 2008).
157 Stepień, M. Malaria in Poland in 2008. (Department of Epidemiology, National Institute of Public Health - National Institute of Hygiene, Warsaw, 2009).
158 Stepień, M. Malaria in Poland in 2009. (Department of Epidemiology, National Institute of Public Health - National Institute of Hygiene, Warsaw, 2010).
159 Stepień, M. Malaria in Poland in 2010. (Department of Epidemiology, National Institute of Public Health - National Institute of Hygiene, Warsaw, 2011).
160 Antunes, F. *et al.* Malaria in Portugal 1977-1986. *Trans R Soc Trop Med Hyg* **81**, 561-562 (1987).
161 Santos, L. C. *et al.* Severe imported malaria in an intensive care unit: a review of 59 cases. *Malar J* **11**, 96, doi:10.1186/1475-2875-11-96 (2012).
162 Castro, L., Cardoso, A. I., Queiros, L. & Goncalves, G. Malaria in Northern Portugal (1993 - 2002) Epidemiological Characterization. *Acta Med Port* **17**, 291-298 (2004).
163 Freira, S. *et al.* Admission for Imported Malaria in Children, Two Hospitals in the Greater Lisbon. *Portuguese Society of Paediatrics* **40**, 65-68 (2009).
164 Baio, P. A. *The clinical knowledge and the importance of malaria epidemic in the non-endemic: future prospects for Europe*, (2011).
165 Fonseca, A. G., Dias, S. S., Baptista, J. L. & Torgal, J. Imported malaria in portugal 2000-2009: a role for hospital statistics for better estimates and surveillance. *Malaria research and treatment* **2014**, 373029, doi:10.1155/2014/373029 (2014).
166 Directorate General of Health. Notifiable Diseases 2002-2006. (2007).
167 Fonseca, A. G., Dias, S. S., Baptista, J. L. & Torgal, J. The burden of imported malaria in Portugal 2003 to 2012. *Journal of travel medicine* **21**, 354-356, doi:10.1111/jtm.12141 (2014).
168 Al-Kuwari, M. G. Epidemiology of Imported Malaria in Qatar. *Journal of travel medicine* **16**, 119-122, doi:10.1111/j.1708-8305.2008.00285.x (2009).
169 Disease Control & Prevention. Malaria Situation in Qatar, 2000/2001. (Department of Ministry of Preventive Health, MOPH-State of Qatar, 2002).
170 D'Ortenzio, E., Sissoko, D., Dehecq, J. S., Renault, P. & Filleul, L. Malaria imported into Réunion Island: is there a risk of re-emergence of the disease? *Transactions of the Royal Society of Tropical Medicine and Hygiene* **104**, 251-254, doi:10.1016/j.trstmh.2009.10.008 (2010).
171 Neghina, R., Neghina, A. M., Giurgiu, L. D., Marincu, I. & Iacobiciu, I. Import of malaria in a Romanian Western County. *Travel Med Infect Dis* **6**, 215-218, doi:10.1016/j.tmaid.2007.12.001 (2008).
172 Neghina, R., Neghina, A. M. & Iacobiciu, I. Malaria in Romania and Its Relationship to International Travel. *Infectious Diseases in Clinical Practice* **18**, 159-161 (2010).
173 Neghina, R., Neghina, A. M., Marincu, I. & Iacobiciu, I. International travel increase and malaria importation in Romania, 2008-2009. *Vector borne and zoonotic diseases* **11**, 1285-1288, doi:10.1089/vbz.2010.0275 (2011).
174 Parasca, L., Grecu, S. & Miron, L. International travel increase and malaria importation in Romania, 2007-2012. *Lucrări Ştiinţifice-Medicină Veterinară, Universitatea de Ştiinţe Agricole şi Medicină Veterinară" Ion Ionescu de la Brad" Iaşi* **56**, 330-339 (2013).

175 Neghina, R. *et al.* Two cases of imported malaria in Western Romania, 2010-2011. *Asian Pacific journal of tropical medicine* **5**, 326-328, doi:10.1016/S1995-7645(12)60049-2 (2012).
176 Alghamdi, A. H., Ibrahim, A. M. & Alghamdi, M. Pattern of National and International Importation of Malaria Infection: Jeddah Province, Saudi Arabia. *International Journal of Health Research and Innovation* **2**, 61-68 (2014).
177 Unger, H. W. *et al.* Imported malaria in Scotland--an overview of surveillance, reporting and trends. *Travel Med Infect Dis* **9**, 289-297, doi:10.1016/j.tmaid.2011.10.001 (2011).
178 Dakic, Z. *et al.* Imported malaria in Belgrade, Serbia, between 2001 and 2009. *Wien Klin Wochenschr* **123 Suppl 1**, 15-19, doi:10.1007/s00508-011-0040-x (2011).
179 Batut, M. J. Report about communicable diseases in 2011 in the territory of Serbia. (Institute for Public Health of Serbia, Centre for Disease Prevention and Control, 2012).
180 Batut, M. J. Report about communicable diseases in 2012 in the territory of Serbia. (Institute for Public Health of Serbia, Centre for Disease Prevention and Control, 2013).
181 Lee, Y. C. A. *et al.* Epidemiological Characteristics of Imported and Locally-acquired Malaria in Singapore. *Annals of the Academy of Medicine* **38**, 840-849 (2009).
182 Svihrova, V., Szilagyiova, M., Novakova, E., Svihra, J. & Hudeckova, H. Costs analysis of the treatment of imported malaria. *Malar J* **11**, 1, doi:10.1186/1475-2875-11-1 (2012).
183 Svihrova, V. *et al.* Analysis of the direct and indirect costs of treatment of imported malaria in the Slovak Republic. *Rev Soc Bras Med Trop* **42**, 377-380 (2009).
184 Regionálny úrad verejného zdravotníctva so sídlom v Banskej Bystrici. Health Yearbook of Banská Bystrica and Brezno in 2012. (2013).
185 Ministry of Health of the Republic of Slovenia & Institute for Public Health of the Republic of Slovenia. Epidemiological surveillance of communicable diseases in Slovenia (2000-2011). (2012).
186 Šubelj, M. & Sočan, M. Imported malaria in Slovenia, 2001–2011. *Central European Journal of Medicine* **7**, 290-295, doi:10.2478/s11536-011-0159-1 (2012).
187 López-Vélez, R., Huerga, H. & Turrientes, M. C. Infectious diseases in immigrants from the perspective of a tropical medicine referral unit. *Am J Trop Med Hyg* **69**, 115-121 (2003).
188 López-Vélez, R. *et al.* Clinicoepidemiological study of imported malaria in travelers and immigrants to Madrid. *Journal of travel medicine* **6**, 81-86 (1999).
189 Millet, J. P. *et al.* Imported malaria in a cosmopolitan European city: a mirror image of the world epidemiological situation. *Malar J* **7**, 56, doi:10.1186/1475-2875-7-56 (2008).
190 Millet, J. P. *et al.* Imported malaria among African immigrants: is there still a relationship between developed countries and their ex-colonies? *Malar J* **8**, 111, doi:10.1186/1475-2875-8-111 (2009).
191 Monge-Maillo, B. *et al.* Imported infectious diseases in mobile populations, Spain. *Emerging infectious diseases* **15**, 1745-1752, doi:10.3201/eid1511.090718 (2009).
192 Ministerio de sanidad y consumo. Malaria in Spain 1989-2006. (2007).
193 Huerga, H. & Lopez-Velez, R. Imported malaria in immigrant and travelling children in Madrid. *European journal of clinical microbiology & infectious diseases : official publication of the European Society of Clinical Microbiology* **20**, 591-593 (2001).
194 Garcia-Villarrubia, M. *et al.* Epidemiology of imported malaria among children and young adults in Barcelona (1990-2008). *Malar J* **10**, 347, doi:10.1186/1475-2875-10-347 (2011).
195 González, A. *et al.* Severe Imported Malaria in Adults: Retrospective Study of 20 Cases. *American Journal of Tropical Medicine and Hygiene* **81**, 595-599, doi:10.4269/ajtmh.2009.08-0637 (2009).
196 Cervera Miguel, J. I., Navarro Ibanez, V., Calabuig Munoz, E. & Peman Garcia, J. [Malaria. Analysis of 31 cases (1993-2002)]. *Revista clinica espanola* **204**, 317-319 (2004).
197 Espinosa-Vega, E. *et al.* Malaria in Paradise: Characterization of Imported Cases in Gran Canaria Island (1993-2006). *Journal of travel medicine* **18**, 165-172, doi:10.1111/j.1708-8305.2011.00503.x (2011).

198 Arnáez, J. *et al.* Imported malaria in children: a comparative study between recent immigrants and immigrant travelers (VFRs). *Journal of travel medicine* **17**, 221-227, doi:10.1111/j.1708-8305.2010.00416.x (2010).
199 Jiménez, B. C. *et al.* Imported malaria in pregnancy in Madrid. *Malar J* **11**, 112, doi:10.1186/1475-2875-11-112 (2012).
200 Rey, S., Zuza, I., Martinez-Mondejar, B., Rubio, J. M. & Merino, F. J. Imported malaria in an area in southern Madrid, 2005-2008. *Malar J* **9**, 290, doi:10.1186/1475-2875-9-290 (2010).
201 Epidemiological Surveillance Service. Comentario epidemiológico de las Enfermedades de Declaración Obligatoria y Sistema de Información Microbiológica. España. Año 2005-2012. (National Epidemiology Center, Carlos III Health Institute, 2013).
202 López-Vélez, R., Pérez Molina, J. A., Fuertes, P. Z. & de Ayala Balzola, A. P. Enfermedades infecciosas importadas por viajeros internacionales a los trópicos. Report No. 1195-1982 (Print),1195-1982 (Linking), (Ministry of Health and Consumer, 2008).
203 Askling, H. H., Nilsson, J., Tegnell, A., Janzon, R. & Ekdahl, K. Malaria risk in travelers. *Emerging infectious diseases* **11**, 436-441, doi:10.3201/eid1103.040677 (2005).
204 Public Health Agency of Sweden. (2014).
205 Swedish Institute for Communicable Disease Control (Smittskyddsinstitutet). (2014).
206 Nüesch, R., Scheller, M. & Gyr, N. Hospital admissions for malaria in Basel, Switzerland: an epidemiological review of 150 cases. *Journal of travel medicine* **7**, 95-97 (2000).
207 D'Acremont, V., Landry, P., Mueller, I., Pecoud, A. & Genton, B. Clinical and laboratory predictors of imported malaria in an outpatient setting: an aid to medical decision making in returning travelers with fever. *Am J Trop Med Hyg* **66**, 481-486 (2002).
208 D'Acremont, V. *et al.* Treatment of imported malaria in an ambulatory setting: prospective study. *Bmj* **324**, 875-877 (2002).
209 Thierfelder, C., Schill, C., Hatz, C. & Nuesch, R. Trends in imported malaria to Basel, Switzerland. *Journal of travel medicine* **15**, 432-436, doi:10.1111/j.1708-8305.2008.00251.x (2008).
210 Bochatay, L., Sudre, P., Chappuis, F., Le Lin, B. & Loutan, L. [Imported malaria in Geneva: 1998-2004]. *Revue medicale suisse* **2**, 1256-1258, 1260-1251 (2006).
211 Eidgenossisches Department des Innern EDI. Malaria: Reported cases in Switzerland from 2003 to 2005 and overview of the international situation. (Switzerland, 2007).
212 Fenner, L., Weber, R., Steffen, R. & Schlagenhauf, P. Imported infectious disease and purpose of travel, Switzerland. *Emerging infectious diseases* **13**, 217-222, doi:10.3201/eid1302.060847 (2007).
213 Section Epidémiologie et section Vaccinations et mesures de contrôle. Malaria in Switzerland from 2006 to 2010. (2011).
214 Taiwan Provincial Institute of Infectious Diseases. Imported malaria among travelers, 1981-1984. (1985).
215 Chadee, D. D. & Kitron, U. Spatial and temporal patterns of imported malaria cases and local transmission in Trinidad. *Am J Trop Med Hyg* **61**, 513-517 (1999).
216 Williams, J. P., Chitre, M. & Sharland, M. Increasing Plasmodium falciparum malaria in southwest London: a 25 year observational study. *Arch Dis Child* **86**, 428-430 (2002).
217 Bradley, D. J. Current trends in malaria in Britain. *J R Soc Med* **82 Suppl 17**, 8-13 (1989).
218 Bradley, D. J., Warhurst, D. C., Blaze, M., Smith, V. & Williams, J. Malaria imported into the United Kingdom in 1996. *Eurosurveillance* **3**, 40-42, doi:107 [pii] (1998).
219 Health Protection Agency. Malaria prevention – advice to travellers. (London, 2003).
220 Smith, A. D. *et al.* Imported malaria and high risk groups: observational study using UK surveillance data 1987-2006. *Bmj* **337**, a120-a120, doi:10.1136/bmj.a120 (2008).
221 Elawad, B. B. & Ong, E. L. Retrospective study of malaria cases treated in Newcastle General Hospital between 1990 and 1996. *Journal of travel medicine* **5**, 193-197 (1998).

222 Brabin, B. J. & Ganley, Y. Imported malaria in children in the UK. *Arch Dis Child* **77**, 76-81 (1997).
223 Bunn, A., Escombe, R., Armstrong, M., Whitty, C. J. & Doherty, J. F. Falciparum malaria in malaria-naive travellers and African visitors. *QJM* **97**, 645-649, doi:10.1093/qjmed/hch113 (2004).
224 Harling, R. *et al.* Burden and cost of imported infections admitted to infectious diseases units in England and Wales in 1998 and 1999. *Journal of Infection* **48**, 139-144, doi:10.1016/s0163-4453(03)00080-x (2004).
225 Health Protection Agency. Who is at risk of imported malaria? , (PHLS Communicable Disease Surveillance Centre, London, 2002).
226 Ladhani, S., Aibara, R. J., Blaze, M., Smith, V. & Shingadia, D. V. Trends in imported childhood malaria in the UK: 1999-2003. *Arch Dis Child* **91**, 911-914, doi:10.1136/adc.2005.089433 (2006).
227 Moore, D. A., Grant, A. D., Armstrong, M., Stumpfle, R. & Behrens, R. H. Risk factors for malaria in UK travellers. *Trans R Soc Trop Med Hyg* **98**, 55-63 (2004).
228 Health Protection Agency. Statistics on imported malaria for the year 2001. (London, 2002).
229 Health Protection Agency. Malaria imported into the United Kingdom in 2004: implications for those advising travellers (London, 2005).
230 Health Protection Agency. Malaria imported into the United Kingdom in 2005: implications for those advising travellers. (London, 2006).
231 Health Protection Agency. Malaria imported into the United Kingdom in 2006: Implications for those advising travellers. (London, 2007).
232 Health Protection Agency. Malaria imported into the United Kingdom in 2007: Implications for those advising travellers. (London, 2008).
233 Health Protection Agency. Malaria imported into the United Kingdom in 2008: implications for those advising travellers. (London, 2009).
234 Health Protection Agency. Malaria imported into the United Kingdom in 2009: implications for those advising travellers. (London, 2010).
235 Health Protection Agency. Malaria imported into the United Kingdom in 2010: implications for those advising travellers. (London, 2011).
236 Health Protection Agency. Malaria imported into the United Kingdom in 2011: implications for those advising travellers. (London, 2012).
237 Health Protection Agency. Malaria imported into the United Kingdom in 2012: implications for those advising travellers. (London, 2013).
238 Health Protection Agency. Malaria imported into the United Kingdom in 2013: implications for those advising travellers. (London, 2014).
239 Health Protection Agency. Malaria imported into the United Kingdom in 2014: implications for those advising travellers. (London, 2015).
240 Lesko, C., Arguin, P. & Newman, R. Congenital Malaria in the United States A Review of Cases From 1966 to 2005. *ARCH PEDIATR ADOLESC MED* **161**, 1062-1067 (2007).
241 Filler, S. *et al.* Malaria Surveillance — United States, 2001. (Centers for Disease Control and Prevention, 2003).
242 Causer, L. M. *et al.* Malaria Surveillance — United States, 2000. (2002).
243 Shah, S. *et al.* Malaria Surveillance — United States, 2002. (Centers for Disease Control and Prevention, 2004).
244 Eliades, M. J. *et al.* Malaria Surveillance — United States, 2003. (Centers for Disease Control and Prevention, 2005).
245 Skarbinski, J. *et al.* Malaria Surveillance — United States, 2004. (Centers for Disease Control and Prevention, 2006).
246 Thwing, J. *et al.* Malaria Surveillance — United States, 2005. (Centers for Disease Control and Prevention, 2007).

247 Mali, S., Steele, S., Slutsker, L. & Arguin, P. M. Malaria Surveillance — United States, 2006. (Centers for Disease Control and Prevention 2008).
248 Mali, S., Steele, S., Slutsker, L. & Arguin, P. M. Malaria Surveillance — United States, 2007. (Centers for Disease Control and Prevention, 2009).
249 Mali, S., Steele, S., Slutsker, L. & Arguin, P. M. Malaria Surveillance — United States, 2008. (Centers for Disease Control and Prevention, 2010).
250 Cullen, K. A. & Arguin, P. M. Malaria Surveillance — United States, 2012. 1-22 (Centers for Disease Control Prevention, 2014).
251 Florida Department of Health. Malaria surveillance--Florida, 2009. (2010).
252 Mali, S. *et al.* Malaria surveillance — United States, 2009. *Morbidity and mortality weekly report. Surveillance summaries* **60**, 1-15 (2011).
253 Florida Department of Health. Malaria surveillance--Florida, 2010. (2011).
254 Mali, S. *et al.* Malaria surveillance — United States, 2010. *Morbidity and mortality weekly report. Surveillance summaries* **61**, 1-17 (2012).
255 Florida Department of Health. Malaria surveillance--Florida, 2011. (2012).
256 Cullen, K. A. & Arguin, P. M. Malaria Surveillance — United States, 2011. 1-17 (Centers for Disease Control Prevention, 2013).
257 Newman MEJ, Girvan M. Finding and evaluating community structure in networks. Phys Rev E 2003; 69(2): 026113.
258 Clauset A, Newman MEJ, Moore C. Finding community structure in very large networks. Phys Rev E 2004; 70(6): 066111.
